# Supplementary material for: Photogenerated outer electric field induced electrophoresis of organic nanocrystals for effective solid-solid photocatalysis
Source: Nat Commun. 2024 Jan 10;15:428. doi: 10.1038/s41467-024-44700-w (PMC10781792; doi:10.1038/s41467-024-44700-w)
Supplement: Supplementary file 1 — Supplementary Information [file 41467_2024_44700_MOESM1_ESM.pdf]

Supplementary Information for

Photogenerated outer electric field induced electrophoresis of organic nanocrystals for effective solid-solid photocatalysis

Yan Guo[1,2], Bowen Zhu[3], Chuyang Y. Tang[2*], Qixin Zhou[1]* and Yongfa Zhu[1]*

[1]Department of Chemistry, Tsinghua University, Beijing, 100084, China

[2]Department of Civil Engineering, The University of Hong Kong, Hong Kong, 999077, China

[3]School of Environmental and Energy Engineering, Beijing University of Civil Engineering and Architecture, 100032, Beijing, China

*E-mails: tangc@hku.hk, zqx20@mails.tsinghua.edu.cn, zhuyf@tsinghua.edu.cn

This file includes:

Supplementary Figs. 1 to 39

Supplementary Notes 1-4

Supplementary Tables 1 to 7

Supplementary References (1 to 16)

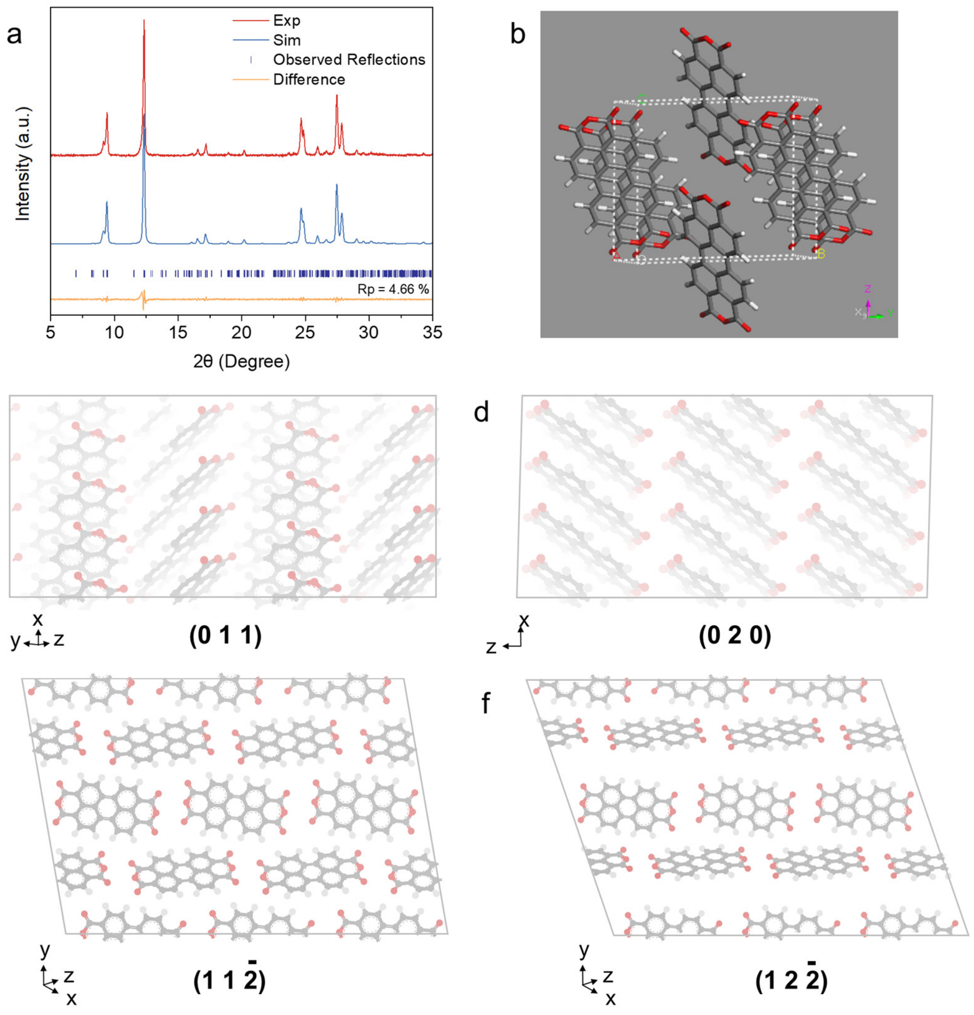

Supplementary Figure 1. Crystal analysis of molecular crystal PTCDA. (a) Measured and simulated powder diffraction of PTCDA molecular crystal. (b) Refined PTCDA molecular crystal model. Crystal facet (c) (0 1 1), (d) (0 2 0), (e) (1 1 $\bar{2}$), (f) (1 2 $\bar{2}$).

Theoretical calculations and simulations align well with experimental powder XRD results. Due to the ordered arrangement of molecules in the crystal, molecular crystals display notable variations in molecular configurations among different facets. The PTCDA crystal show an oxygen-enriched (0 1 1) facet, a hydrogen- enriched (0 2 0) facet, conjugation plane (1 1 $\bar{2}$) and (1 2 $\bar{2}$) facet.

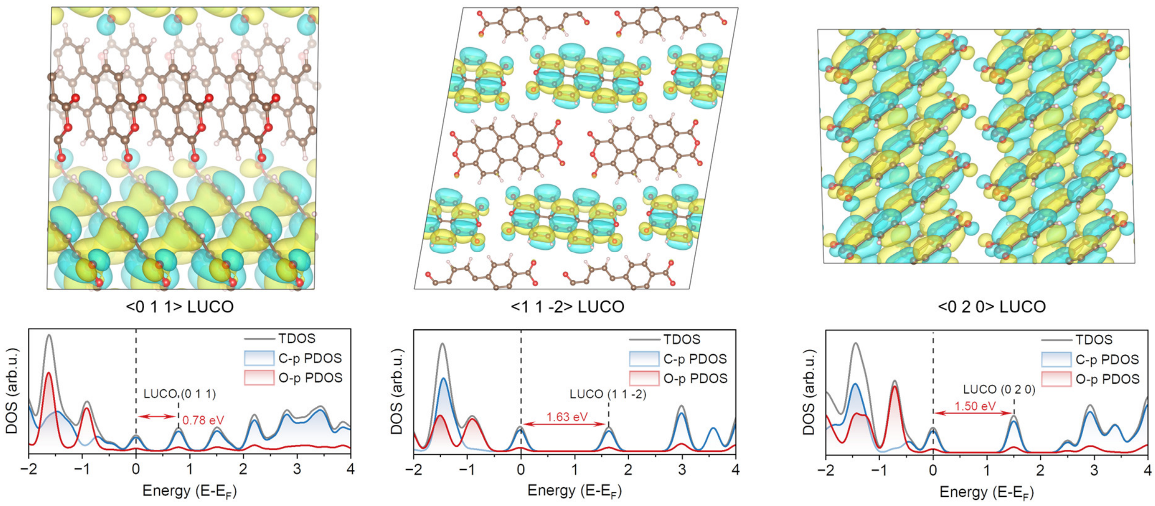

Supplementary Figure 2. Theoretical calculations of the lowest unoccupied crystal orbitals (LUCO) and the density of states (DOS) for the (0 1 1), (1 1 $\bar{2}$) and (0 2 0) crystal facets. The isosurface of the molecular orbital is set at a value of 0.0075.

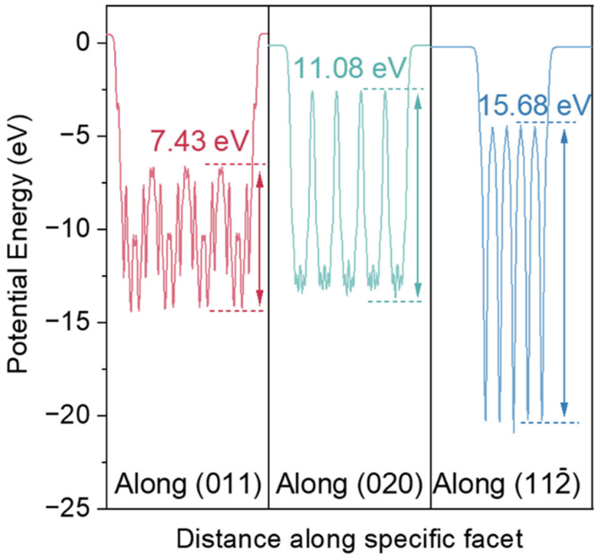

Supplementary Figure 3. The theoretical calculated integral of the electrostatic potential change along the normal direction of (0 1 1), (0 2 0) and (1 1 $\bar{2}$) facet on PTCDA molecular crystals.

The electrostatic potential change integral along the normal direction of three facets was displayed. The smaller the difference, the lower the energy barrier for exciting electron migration, and the more inclined electrons are to migrate. This outcome indicates that photogenerated electrons in the bulk preferentially migrate along the axis of the (0 1 1) facets, accounting for electrons accumulation on (0 1 1) facets.

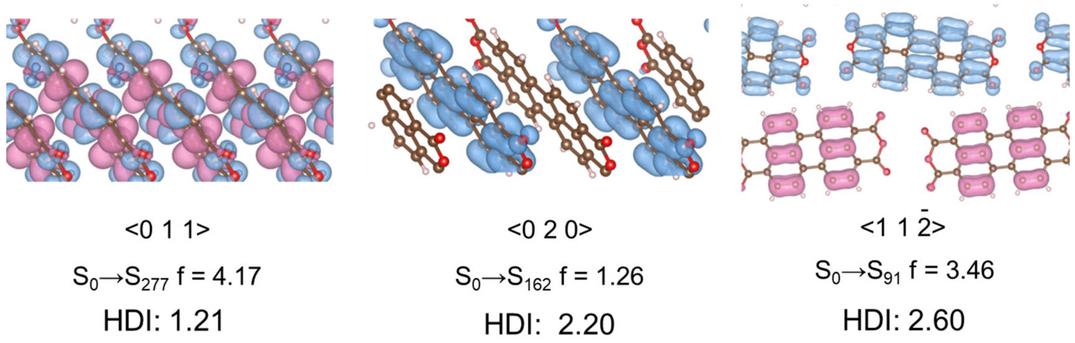

Supplementary Figure 4. Theoretically calculated hole distribution states on the PTCDA crystal surface.

We quantified the distribution of holes on the crystal facet (the thickness of the crystal surface is three layers of molecules, the vacuum layer is 2 nm, and the figure shows the projection perpendicular to the orientation of the facet) in the relaxed state. The red isosurface represents the distribution of electrons in the relaxed state, and the blue isosurface represents the distribution of holes in the relaxed state. In addition to the perspective of energy, photogenerated holes are different from photogenerated electrons generated by direct excitation, and the stable existence of holes on the facet also depends on the relaxation pathway brought by the intrinsic structure. In the (0 1 1) crystal facet, the holes and electrons are tightly bound in the relaxation state, which means that the intrinsic state of the crystal plane has a short-excited state lifetime, so the number of stable holes in the relaxation excitation is reduced. However, the (0 2 0) and $(1\ 1\ \bar{2})$ crystal planes show significant discrete hole distribution (The electrons and holes are distributed on separate molecules), which means that the holes in these crystal faces are mostly lost by external pathways rather than intrinsic relaxation, which provides a structural basis for the long-term stable existence of holes.

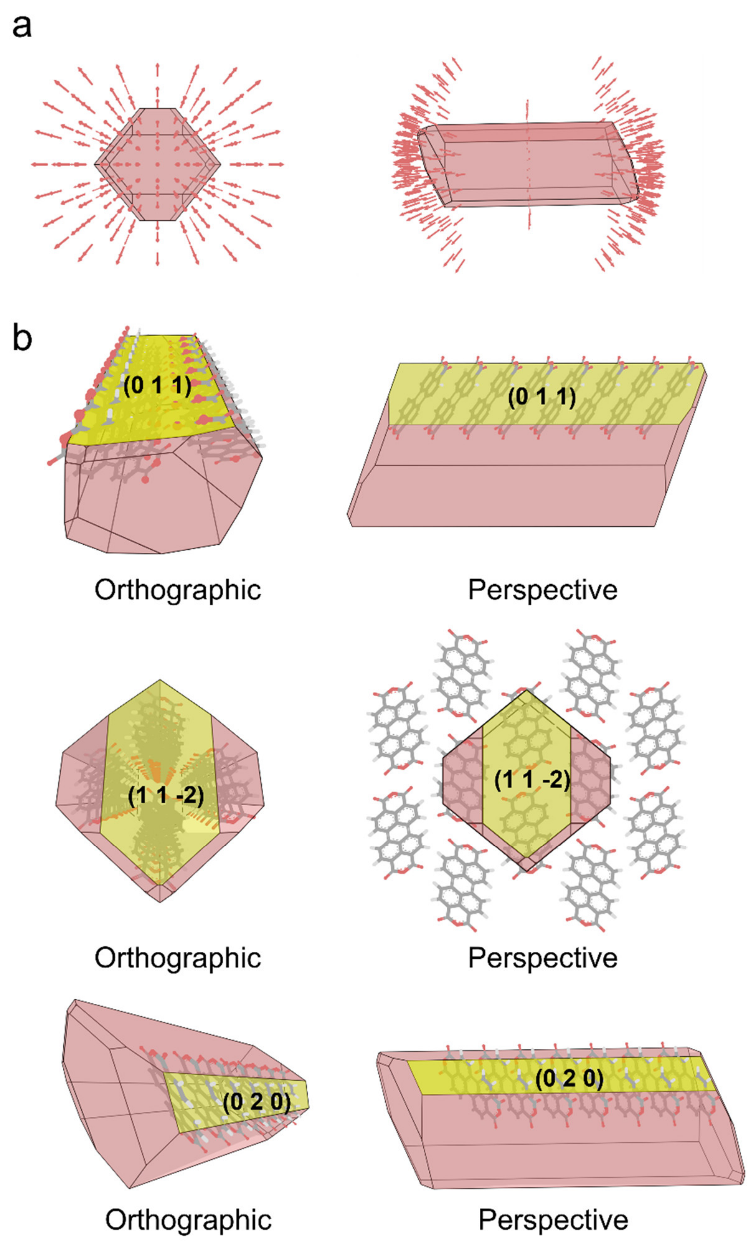

Supplementary Figure 5. Theoretical models of crystal morphology from molecular dynamics predictions. (a) Predicted crystal growth orientation observed from different perspectives. (b) Theoretical model coupled molecular stacking to demonstrate the crystal facet.

Supplementary Figure 5 depicts the theoretical models of crystal morphology, illustrating the growth orientation of PTCDA crystals from various angles. The length of the arrows corresponds

to the degree of crystal growth, with longer arrows indicating more pronounced growth. The predicted outcomes reveal the rod-like growth process of PTCDA crystals. In the simulated PTCDA crystal morphology, the (0 1 1), (1 1 $\bar{2}$) and (0 2 0) planes are identified.

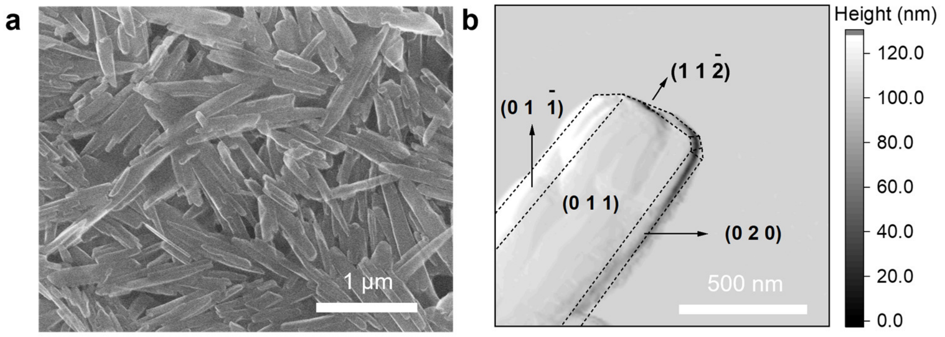

Supplementary Figure 6. Morphology of PTCDA. (a) Scanning transmission electron (SEM) image. (b) Height image of a PTCDA molecular crystal tested by atomic force microscopy (AFM).

The PTCDA molecular crystal exhibits a distinctive rod-like morphology within the monoclinic crystal system. AFM tested the height morphology of PTCDA molecular crystals, and marked the exposed crystal planes according to the model predicted by molecular dynamics.

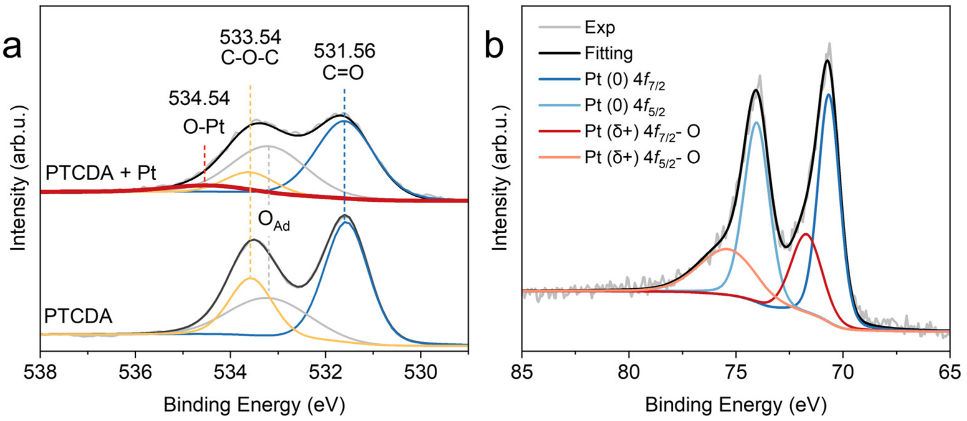

Supplementary Figure 7. X-ray photoelectron spectra (XPS) of O and Pt after photo-deposition of Pt nanoparticle.

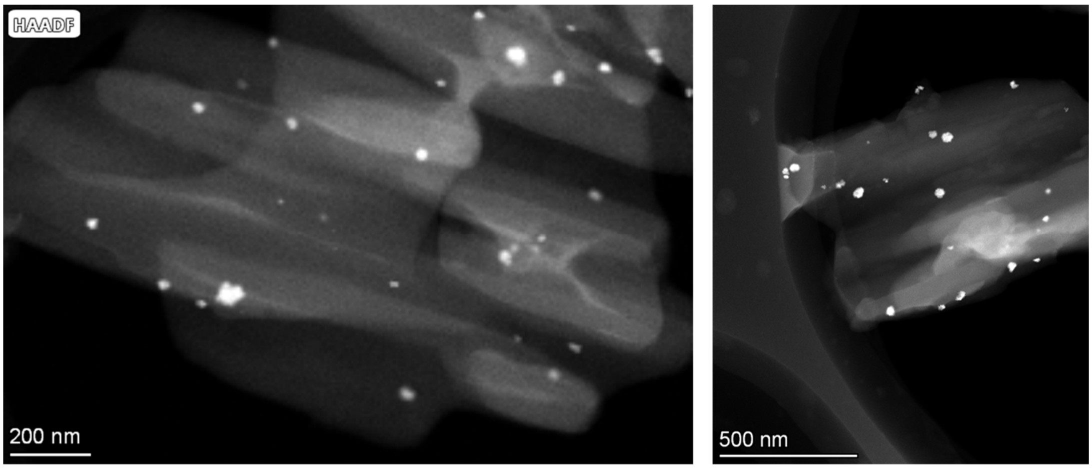

Supplementary Figure 8. High angle annular dark field - scanning transmission electron microscopy (HAADF-STEM) image of PTCDA sample with photo-deposited Pt nanoparticles

In the HAADF-STEM images, the regions with higher brightness typically represent Pt nanoparticles, while those with lower brightness correspond to PTCDA.

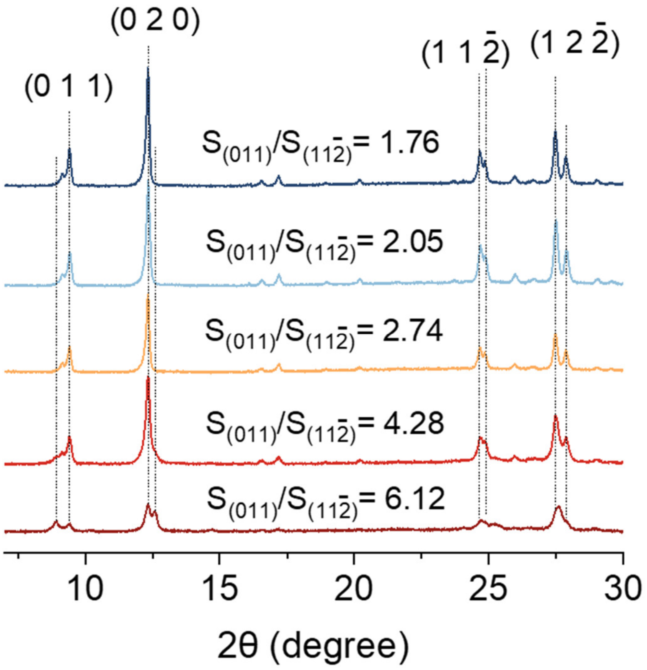

Supplementary Figure 9. Powder X-ray diffraction (PXRD) pattern of PTCDA nanocrystals with different crystal facet ratios. The degree of exfoliation of the PTCDA molecular crystals increases in order from top to bottom.

PXRD patterns demonstrates that the half-peak width of diffraction peaks decreases with a reduction in the degree of exfoliation and an increase in particle size. Simultaneously, a significant splitting of the diffraction peaks at high angles is observed, corresponding to a weaker *π*-*π* interaction within the PTCDA bulk phase [1].

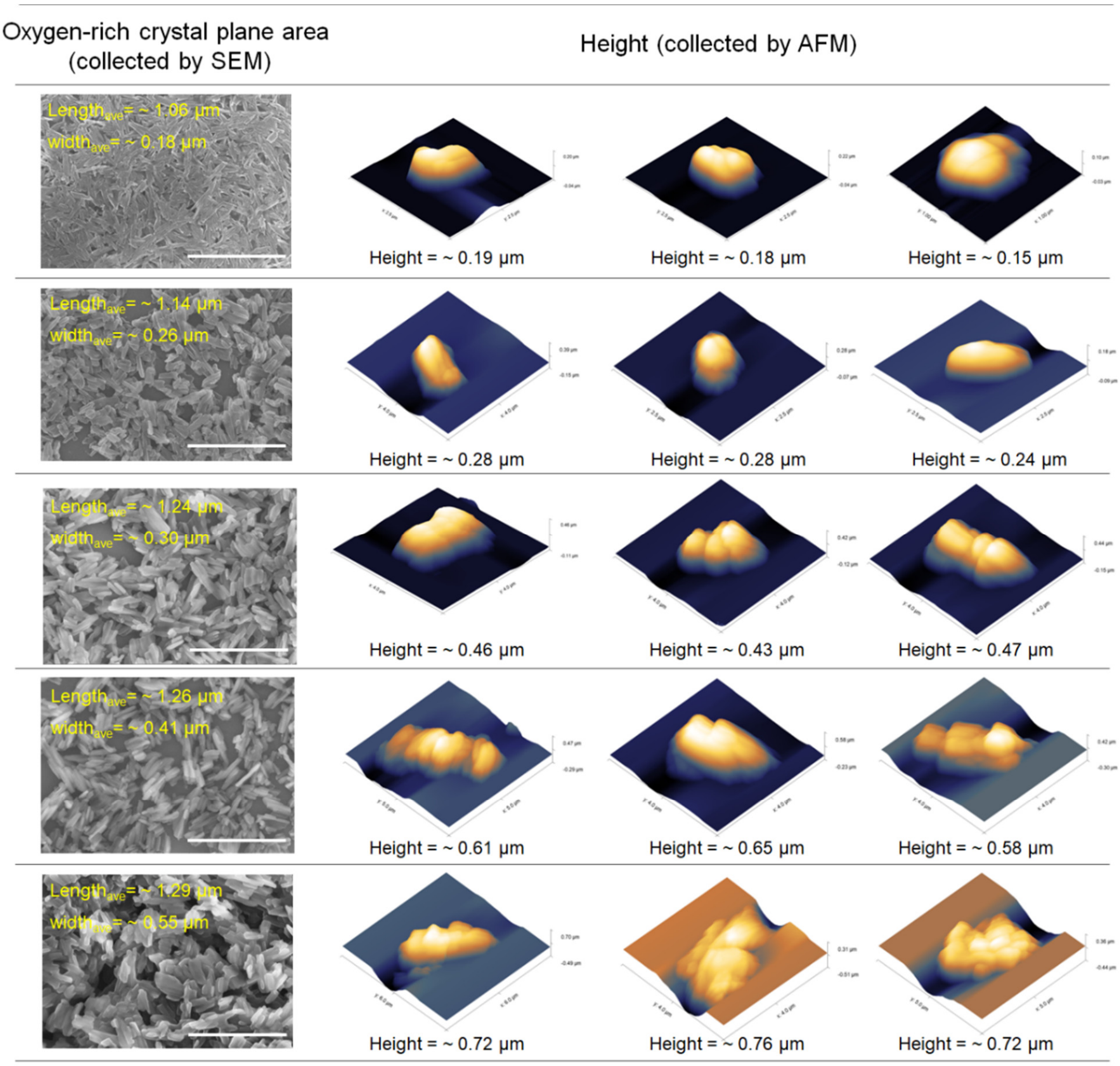

Supplementary Figure 10. Scanning electron micrographs (SEM, left) and atomic force microscopy (AFM, right) of PTCDA molecular crystals modulated by liquid phase exfoliation. The degree of exfoliation of the PTCDA molecular crystals decreases in order from top to bottom.

From top to bottom PTCDA was prepared using 18.4, 9.2, 4.6, 2.3 and 1.2 mol L^{-1} sulphate stripping respectively, as described in METHODS. The elucidated correlation between PTCDA rods and crystal planes allows for the determination of the (0 1 1) and (1 1 $\bar{2}$) facet exposure ratio. The length and width of the PTCDA rods were counted in the SEM image, and the height was

obtained in the AFM image. The exposed areas of the (0 1 1) and (1 1 $\bar{2}$) facet were calculated by the products of length and width, and height and width, respectively.

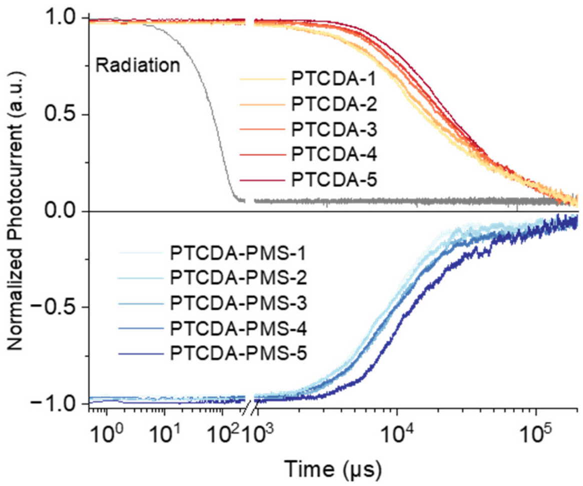

Supplementary Figure 11. Transient photocurrent attenuation of different PTCDA molecular crystals. Numbers 1,2,3,4,5 refer to $S_{(0\ 1\ 1)}$ / $S_{(1\ 1\ \bar{2})}$ of 1.76, 2.05, 2.74, 4.28 and 6.12 respectively.

The instrument defines the cathode current as a positive current, so that the positive current represents the electronic signal generated by the irradiation receiving, and the negative current represents the hole signal generated by the irradiation receiving side.

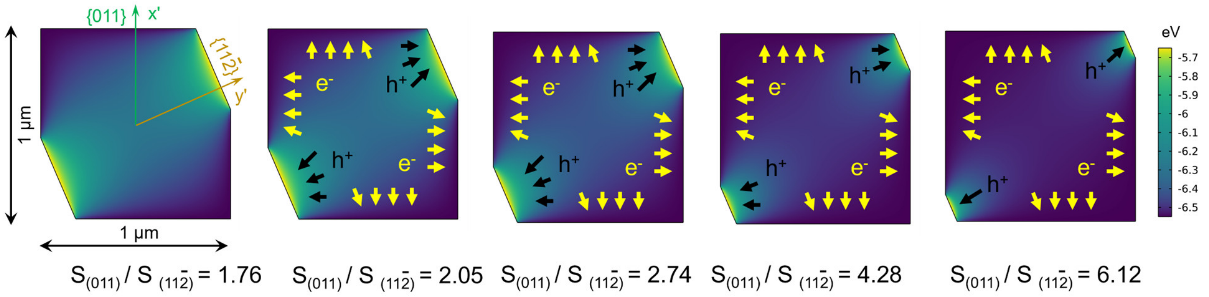

Supplementary Figure 12. Multiscale simulations of photogenerated charge distributions from first-principles extrapolation for PTCDA particles with different $S_{(0\,1\,1)}$ / $S_{(1\,1\,\bar{2})}$.

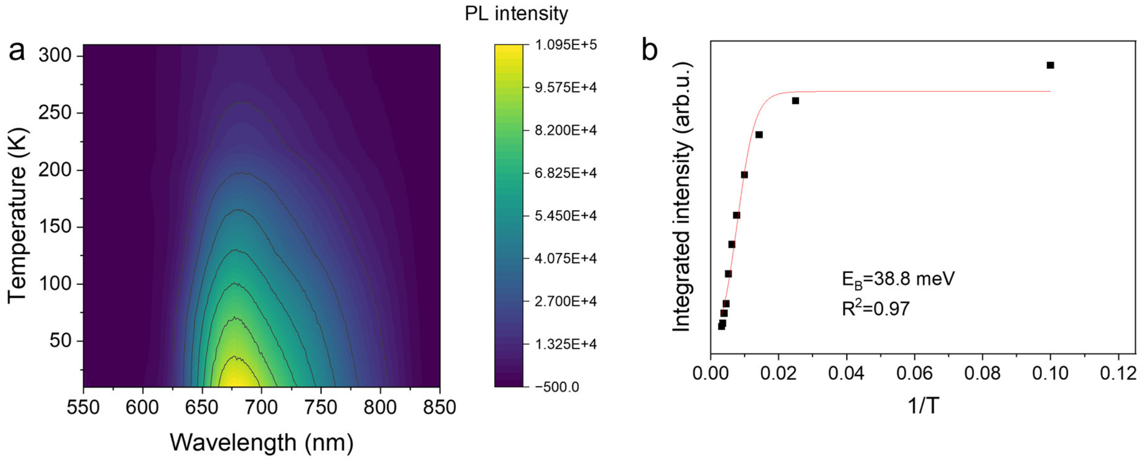

Supplementary Figure 13. Exciton binding energy (E_b) of PTCDA. (a) Temperature-dependent photoluminescence (PL) spectra with excitation wavelength at 400 nm and (b) extracted exciton binding energies of PTCDA.

The exciton binding energy (E_b) is calculated by Equation 1[2, 3]:

$$I(T) = \frac{I_0}{1+Ae^{-E_b/k_BT}} \qquad (1)$$

Where E_b is the binding energy, I_0 is the intensity at 0 K, k_B is the Boltzmann constant, and A is a proportionality constant. Using y=A/(1+B*(exp(C*x))) to fit the parameters in equation 1, an E_b of 38.8 meV was obtained at R^2 of 0.97.

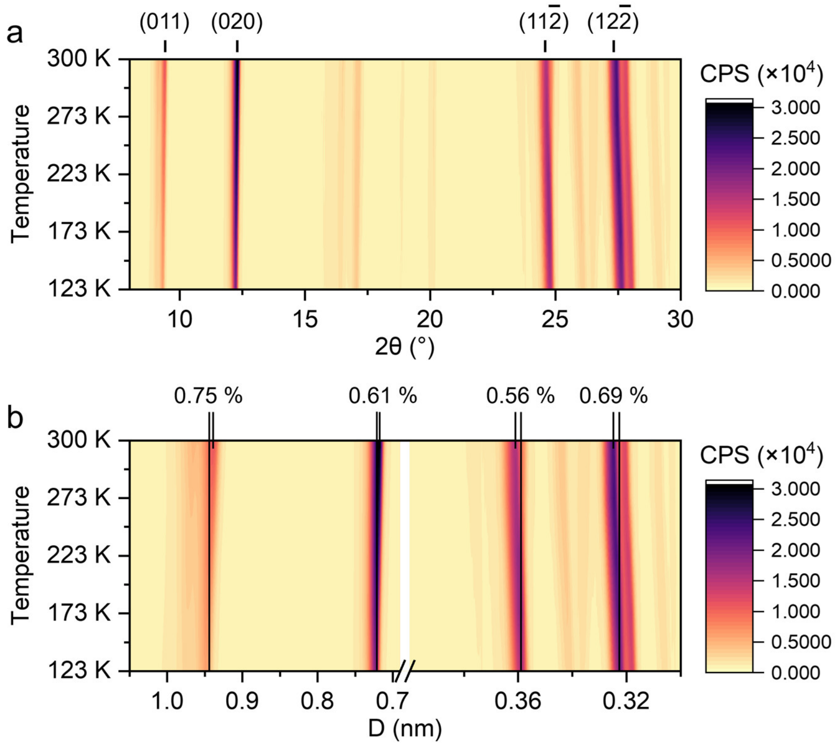

Supplementary Figure 14. Low temperature XRD of PTCDA. (a) Low index diffraction peak changes with temperature, and (b) the corresponding interplanar spacing changes.

The change amplitude of the low-index XRD diffraction peak is within 1%, indicating that the crystal structure has not changed significantly.

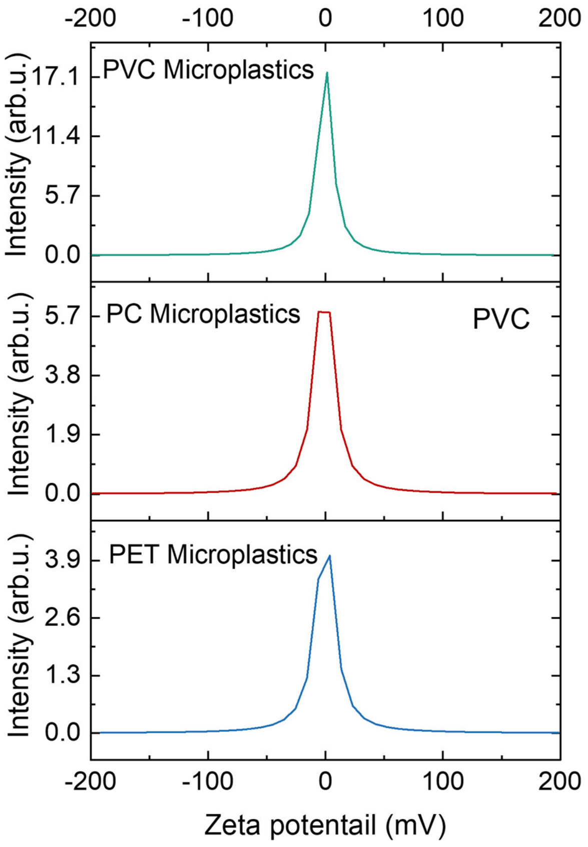

Supplementary Figure 15. Zeta potential of the microplastics used in this study under experimental conditions. The zeta potentials of PVC, PC, and PET in the experimental environments of this work were -0.3 mV, -1.3 mV, and -0.5 mV, respectively.

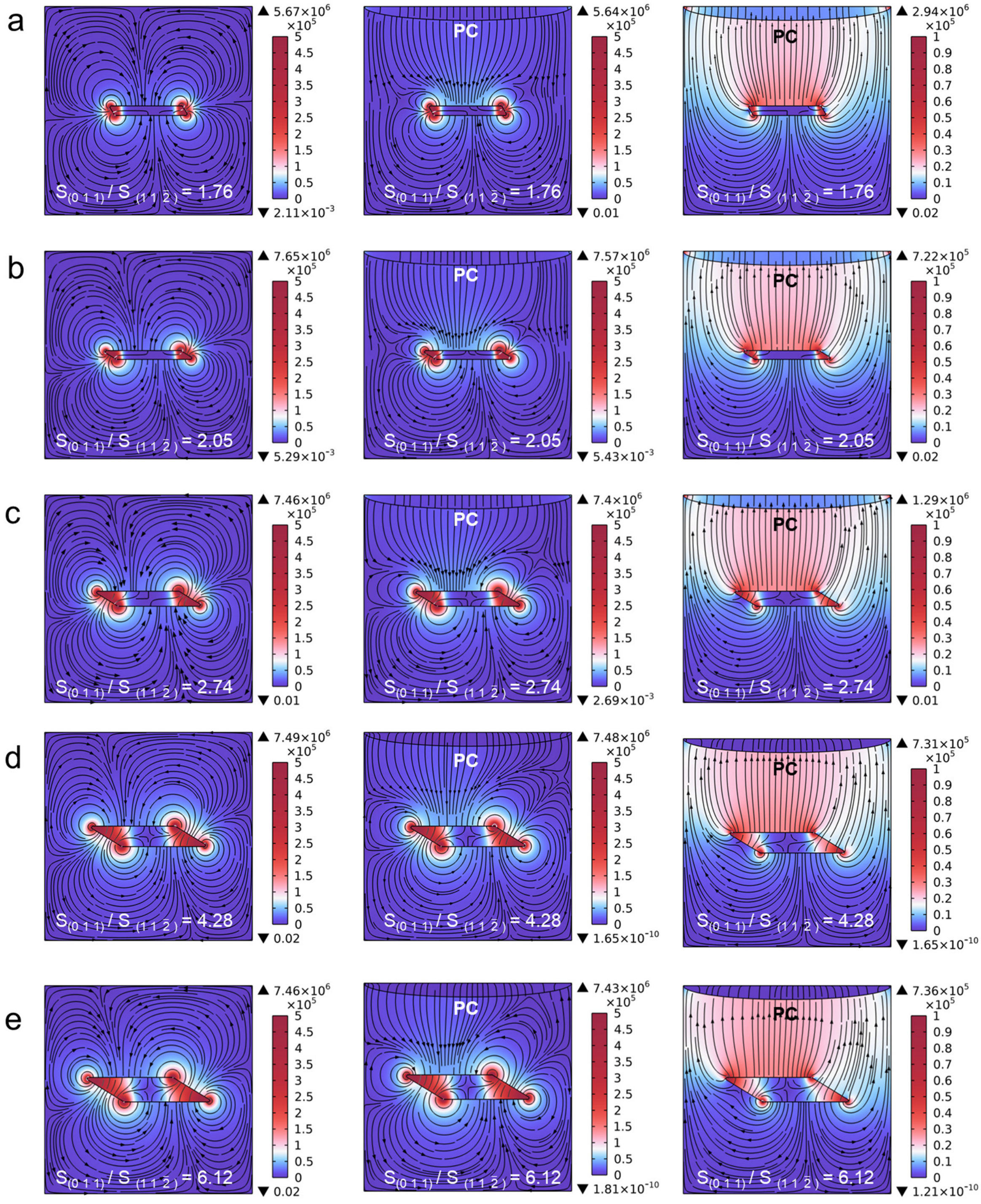

Supplementary Figure 16. Finite element analysis (FEA) of photogenerated external electric field on the surface of PTCDA crystal with (a) 1.76, (b) 2.05, (c) 2.74, (d) 4.28, and (e) 6.12 of $S_{(0\ 1\ 1)}$/ $S_{(1\ 1\ \bar{2})}$. PTCDA crystal surface ratio and charge number are from Supplementary Figures 10, 11,

respectively. The extreme and minima points of the electric field are labeled on the right. Boundary conditions: For the left side, the boundary condition is set at 0 mV. For the middle and right sides, all boundaries are set at 0 mV, except for the upper boundary, which is set at -1.3 mV to simulate the charge on the substrate microplastic surface. Surface charge conditions: For the left and middle sections, no electron acceptor is added. For the right section, the surface is in a hole-occupied state after the addition of an electron acceptor.

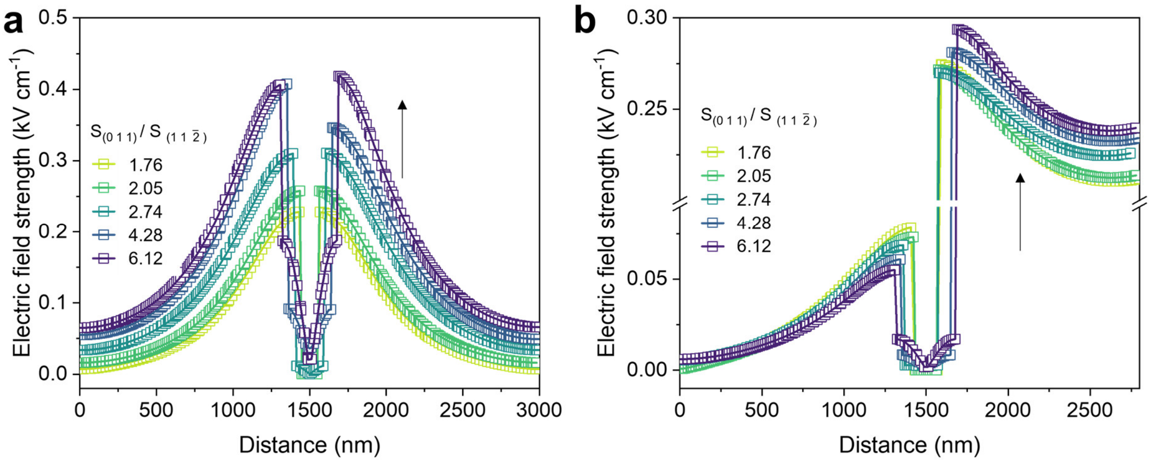

Supplementary Figure 17. The strength of the electric field from the median in the FEA results. (a) initial photogenerated outer electric field (OEF), (b) regulated OEF by electron acceptor PMS.

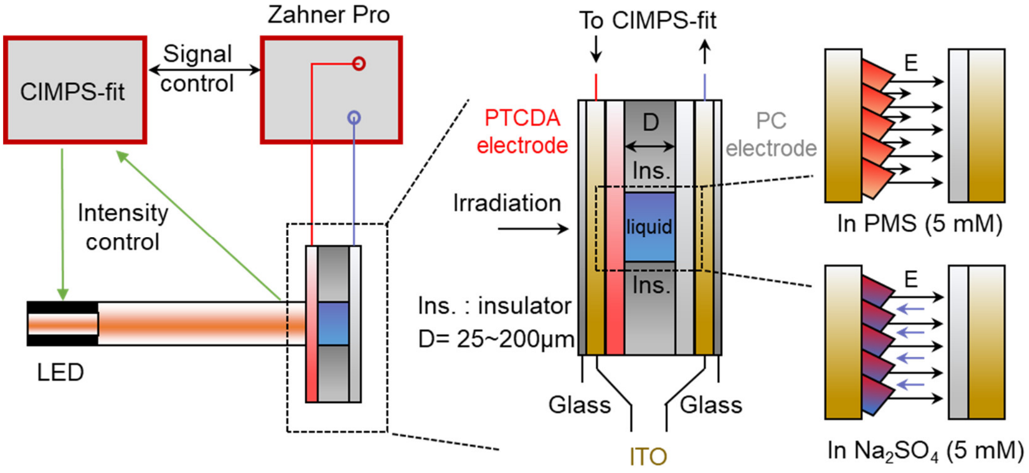

Supplementary Figure 18. Schematic diagram of photogenerated outer electric field (OEF) strength measurement device.

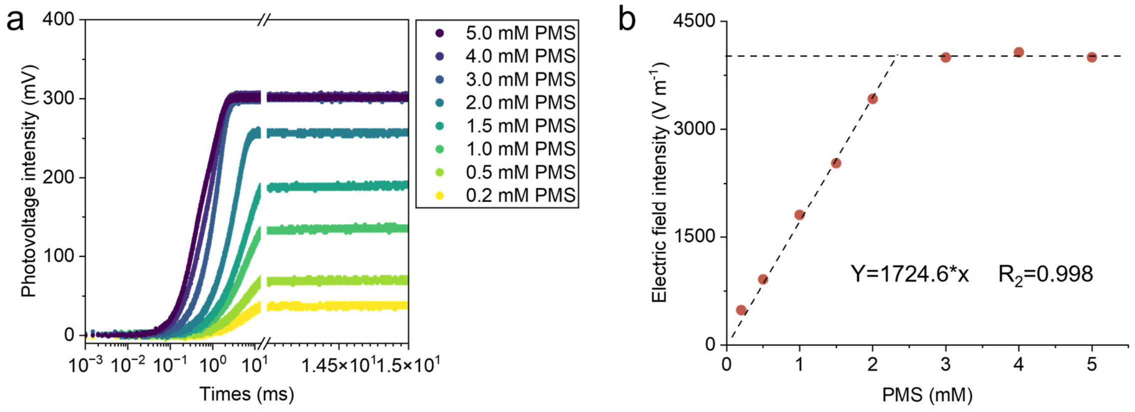

Supplementary Figure 19. The OEF intensity tested for the effect of PMS concentration. (a) Photovoltage intensity as affected by different PMS concentrations. (b) Relationship between PMS concentration and electric field strength. The distance between the PTCDA and PC microplastic electrodes was fixed at 75 μm. The effective working electrode area was 2.25 cm^2.

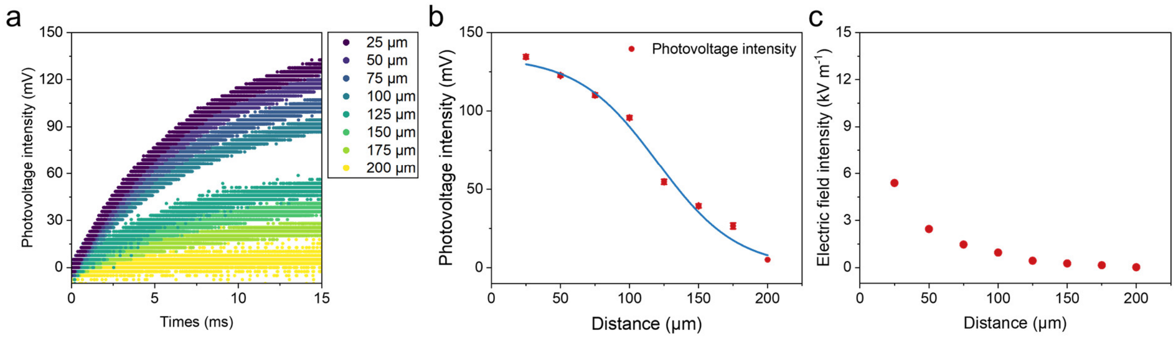

Supplementary Figure 20. Experimentally tested photogenerated potential of PTCDA nanocrystal without adding PMS: (a) point graph of photogenerated voltage over time and distance, (b) balance photogenerated voltage and (c) the corresponding calculated electric field strength.

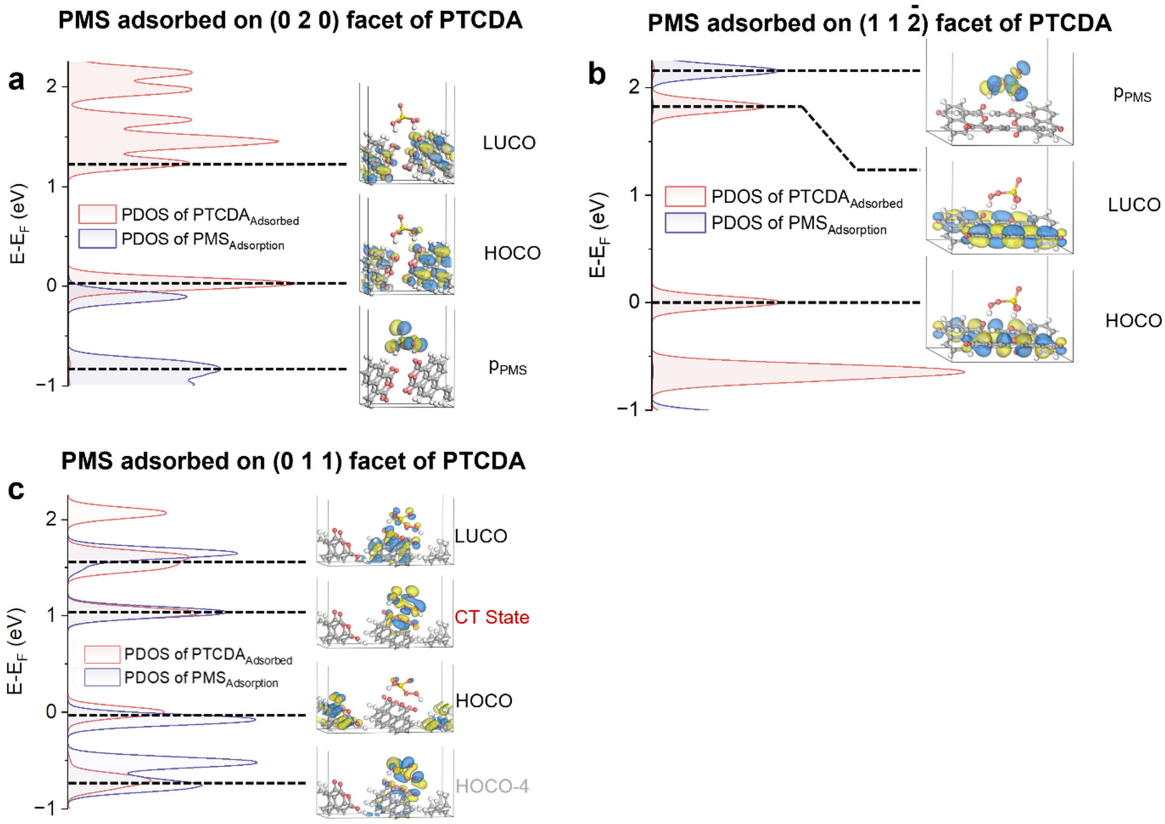

Supplementary Figure 21. Partial Density of States (PDOS) of PMS adsorbed on (a) (0 2 0) facet, (b) (1 1 $\bar{2}$) and (c) (0 1 1) facet of PTCDA.

The PMS hybridizes only with the highest occupied crystal orbital (HOCO) of PTCDA when adsorbed on the (0 2 0) facet. Little hybridization was observed when adsorbed on the (1 1 $\bar{2}$) facet. This indicates that the interaction between PMS and the (0 2 0) and (1 1 $\bar{2}$) facet is weak.

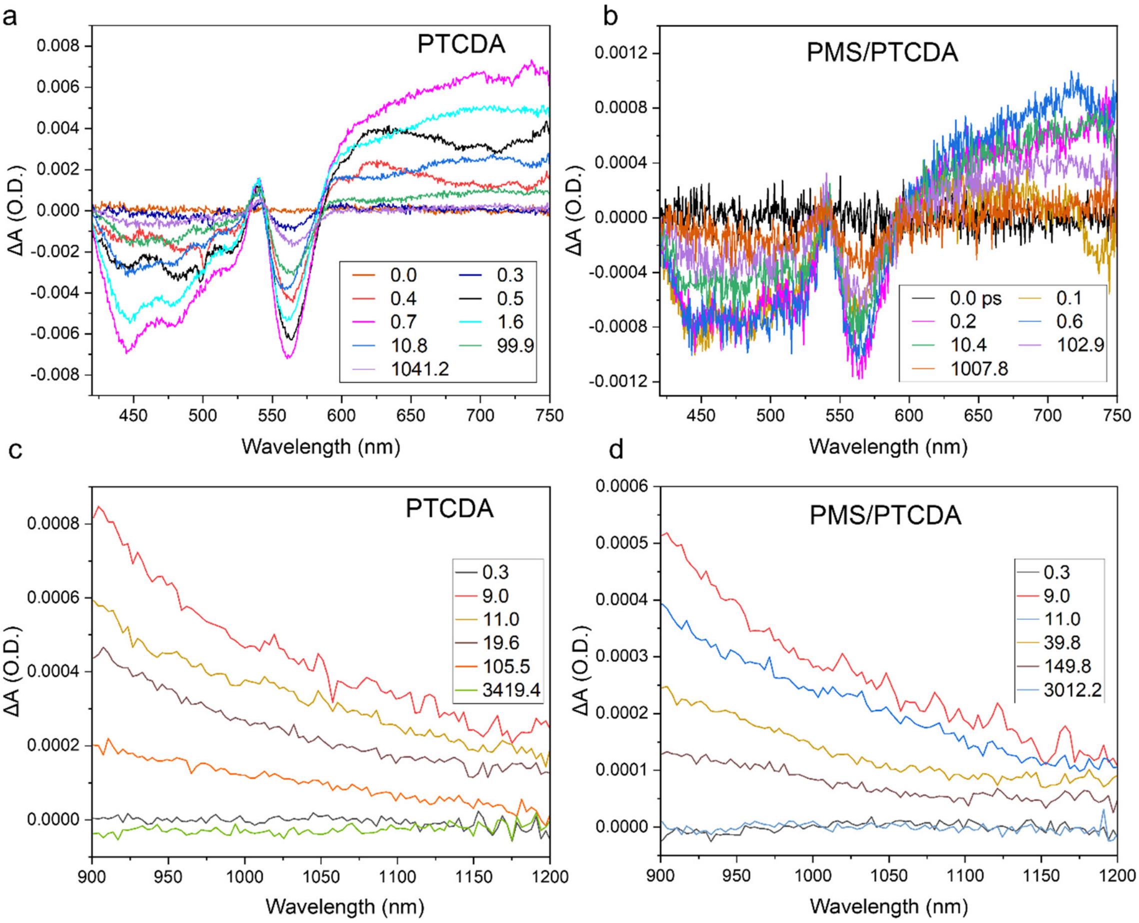

Supplementary Figure 22. Femtosecond transient absorption spectroscopy (fs-TAS) of (a) PTCDA and (b) PMS/PTCDA detected in the visible range. fs-TAS of (c) PTCDA and (d) PMS/PTCDA detected in the near-infrared range. All these data were recorded with a 400 nm laser pulse (37 fs).

Excited state absorption (ESA) of electron and holes is represented by positive absorbance[4, 5]. Near-infrared probing typically detects intraband transitions near the CB level, rendering the 650-1100 nm probe informative for free/shallow trapped electron dynamics [6].The addition of PMS attenuates the absorbance signal since the salting-out effect-induced accelerated sedimentation of PTCDA in suspension. Nevertheless, this does not affect our judgment of the kinetic decay of the absorption peak.

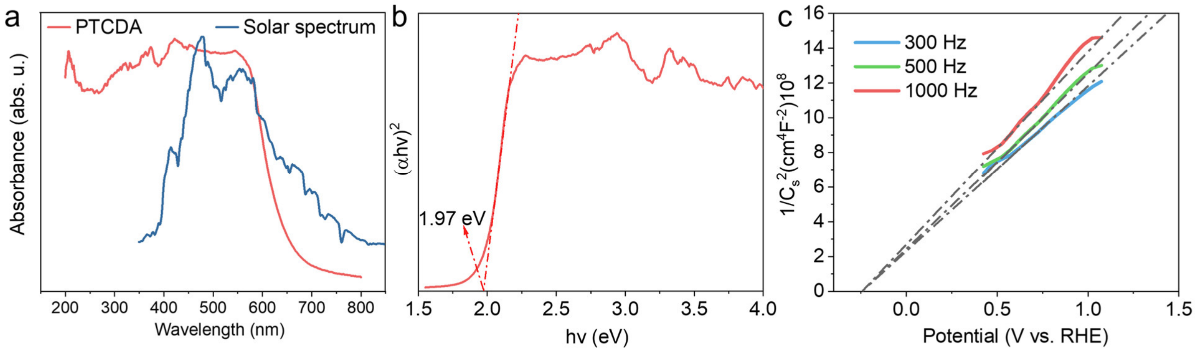

Supplementary Figure 23. The energy band position of PTCDA nanocrystal. (a) UV-vis diffuse reflection spectrum (DRS) of PTCDA and solar spectrum. (b) Plots of the transformed Kubelka–Munk function vs. the light energy. (c) Mott-Schottky plots of PTCDA.

The DRS of PTCDA exhibits a wider absorption at visible light region and the absorption band edge of PTCDA was ~ 629 nm. The band gap derived from the corresponding Tauc plot is 1.97 eV. The Mott-Schottky curve was measured by an electrochemical three-electrode system. PTCDA coated on conductive glass is the working electrode; platinum wire is the counter electrode; Ag/AgCl is the reference electrode; and the electrolyte is a 0.1 M solution of $NaSO_4$. The flat band potential of PTCDA from Mott-Schottky curve is -0.25 V (vs. RHE), respectively. The position of the bottom of the semiconductor conduction band is 0.2 V negative than the flat band potential. Therefore, the conduction band position of PTCDA is -0.45V (vs. RHE), respectively.

Band gap: E_g =1.97 eV,

The position of conduction band (vs. RHE): E_c =-0.45 V

The position of valance band (vs. RHE): $E_v = E_g + E_c = 1.97 + (-0.45) = 1.52$ V.

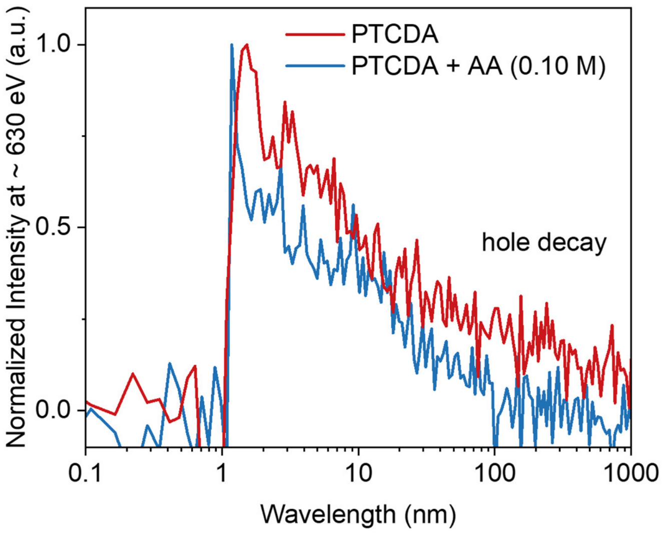

Supplementary Figure 24. The hole quencher ascorbic acid identifies the hole signals of TAS.

The addition of the hole quencher ascorbic acid significantly reduced the delay of the ~630 nm signal, indicating that it is reasonable to analyze the hole kinetics with ~630 nm.

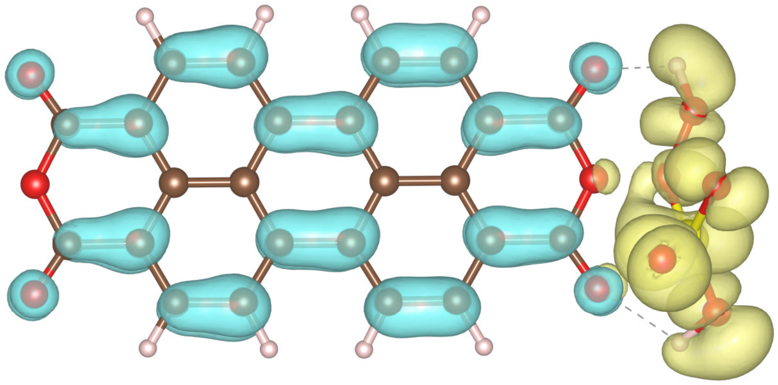

Supplementary Figure 25. The isosurface of the differential charge density by TDDFT (Yellow is charge enriched and blue has reduced electron density).

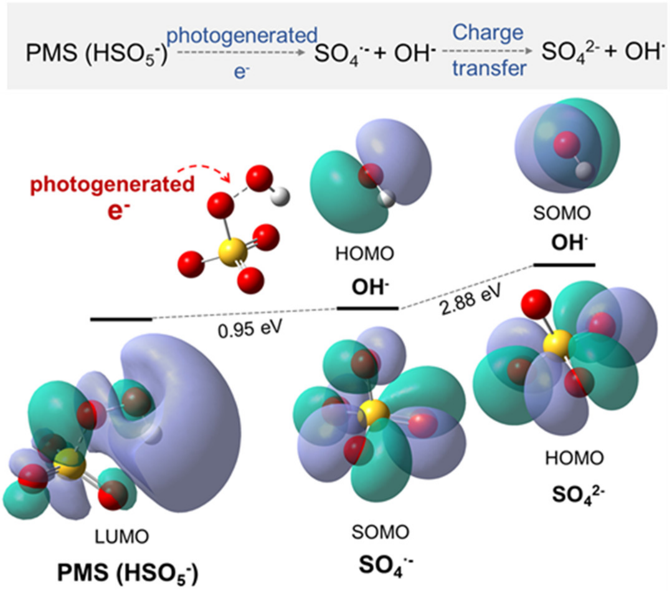

Supplementary Figure 26. Energy calculation for the process of electron capture by the electron acceptor PMS (DFT, Level: M062X/ DEF2TZVP). HOMO: Highest Occupied Molecular Orbital. SOMO: Singly Occupied Molecular Orbital. LUMO: Lowest Unoccupied Molecular Orbital.

The PMS that captures e^- is activated to generate radicals, a process predicted in detail by DFT. 0.95 eV is required for the O-O vacant orbital in the PMS to accept e^-, leading to form $SO_4^{\cdot-}$ and OH^-. Further, the single-electron occupied orbital (SOMO) of $SO_4^{\cdot-}$ accepts OH^- electrons to produce SO_4^{2-} and $OH^{\cdot}$.

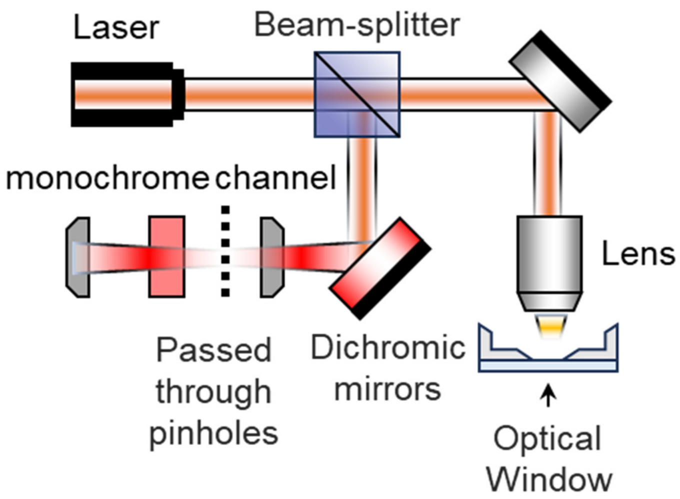

Supplementary Figure 27. Schematic representation of PTCDA nanocrystal movement observed by line scanning confocal reflectance microscopy (LSCRM)

PTCDA exhibits significant fluorescence, enabling observation of its particle behavior through l line scanning confocal reflectance microscopy (LSCRM). PC microplastics were heat-melted and affixed to 20 mm diameter glass-bottomed confocal petri dishes. PTCDA was dispersed in aqueous solutions, either with or without PMS. The petri dish was positioned under a laser confocal microscope, and PTCDA dispersion was added dropwise in situ to monitor migration behavior. A 561 nm low light intensity served as the fluorescence excitation source for PTCDA particles, while a combination of 405 nm, 488 nm, 561 nm, and 640 nm high-intensity light sources functioned as the irradiation source.

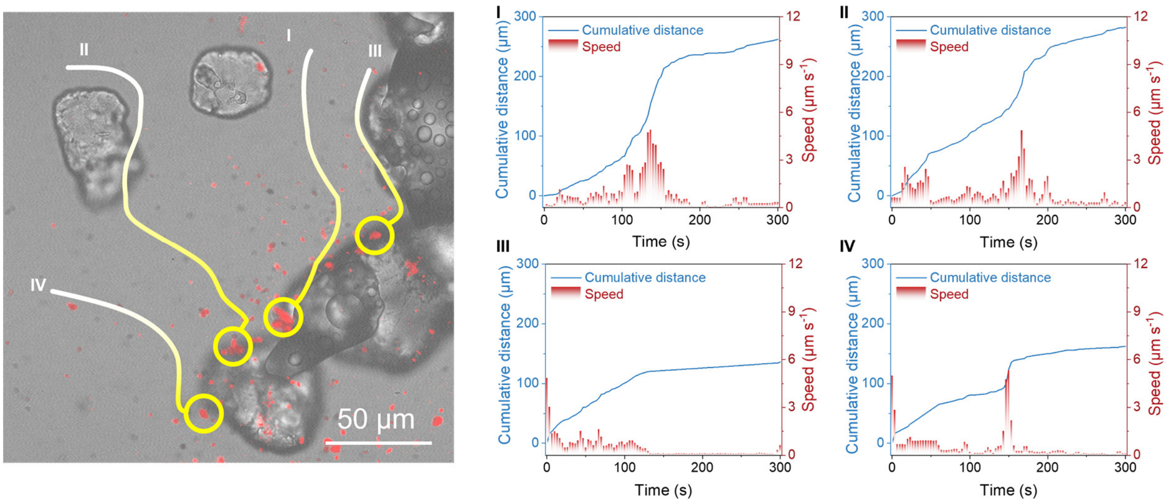

Supplementary Figure 28. Velocity analysis of electrophoretic motion of PTCDA nanocrystals

When the distance between the positively charged PTCDA crystal and the negatively charged microplastic is long (＞20 μm), the surface of the PTCDA crystal has high potential energy and low kinetic energy. Since the photogenerated outer electric field, the nanocrystals begin to approach the surface of the microplastics. In the process of approaching, the potential energy of the particle gradually decreases, while the kinetic energy gradually increases. Therefore, the movement speed of the particles will be gradually accelerated in the early movement (up to 5.4 μm s^{-1}, the coordinates of the nanocrystals are recorded every 3s, and the movement of two adjacent frames is approximately treated as a straight line). However, as the nanocrystals moved closer to the negatively charged plastic, the rate at which the potential energy decreased began to slow, and so did the rate at which the kinetic energy increased. This can be attributed to the fact that the closer the nanocrystals are located to the plastic, the greater the number of negative charges around them, leading to a reduction in the overall effect of the electric field. Although the electric field force between the two charges increases as the distance decreases, the net electric field force on the particle gradually decreases due to the shielding effect of the surrounding negative charges. When

the particles are very close to the plane, the force of the electric field is very weak, and the acceleration of the particles is almost zero. At this time, the velocity of the particles tends to be stable and slows down under the viscosity resistance of the liquid medium. Although the motion process is relatively complex, the average velocity calculated from the four motion trajectories is 0.625 $\mu m\ s^{-1}$.

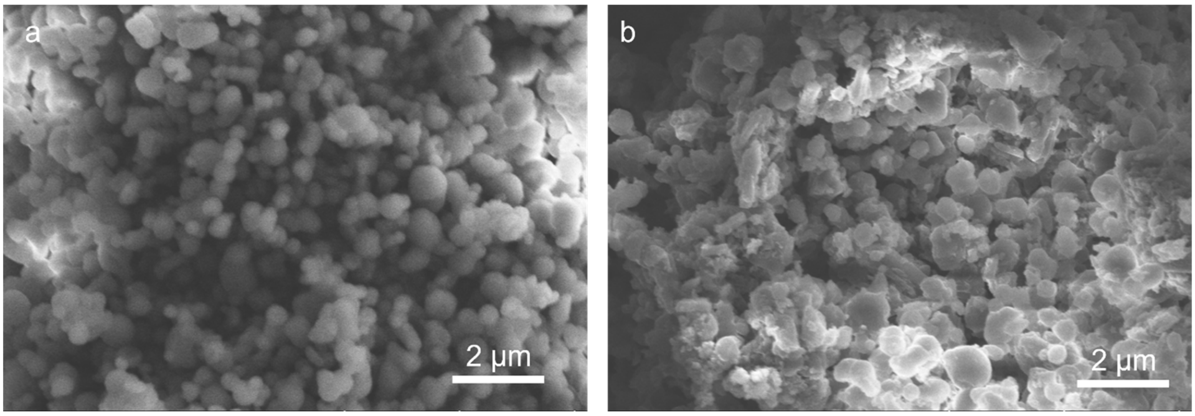

Supplementary Figure 29. SEM images of PC microplastics. (a) Freshly and (b) after 3 h photocatalytic degradation reaction.

Statistics of the microsphere diameters in the SEM field showed that the average particle size of the PC microplastics used in this study was ~500 nm. The particles exhibited full integrity in their pristine state. After 3 hours of photocatalytic reaction, particle integrity was compromised, suggesting progressive decomposition of PC microplastics.

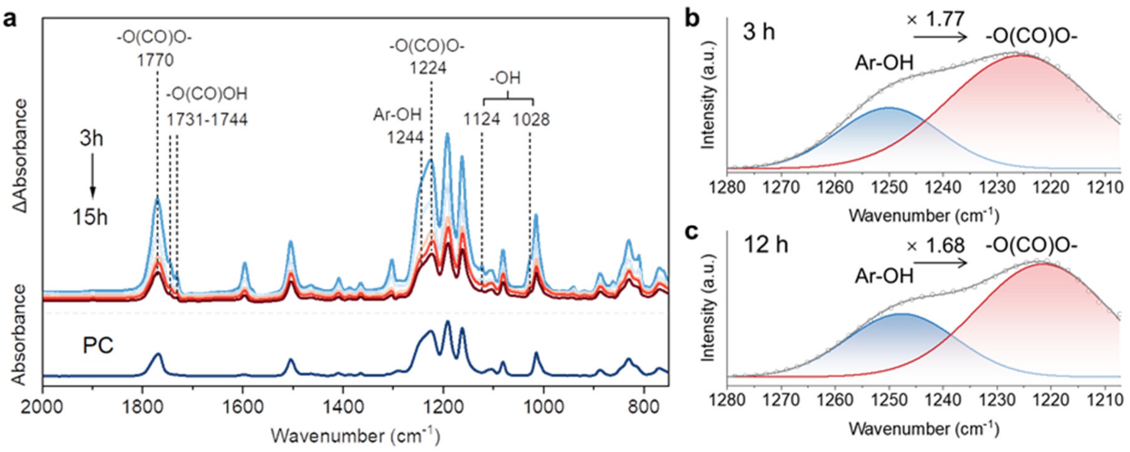

Supplementary Figure 30. Chemical structure and intermediate analysis of solid-phase products. (a) Differential ATR-FTIR measurement of solid-phase species with duration of irradiation. Locally enlarged FTIR, and deconvolution based on Gaussian distribution at (b) 3 h and (c)12 h.

The decomposition process of solid-phase PC microplastics was revealed by the differential spectra of the solid mixture after photocatalytic degradation. New characteristic absorptions appear at 1731-1744 cm^{-1}, 1124 cm^{-1}, and 1028 cm^{-1}, and as the degradation continues, the intensity of these peaks becomes weaker with the overall differential absorption. Based on DFT predictions (b3lyp 6-311+G**em=gd3bj smd solvent=water level), these peaks were attributed to -O(CO)OH generated after -O(CO)O- fragmentation and associated -OH vibration. This feature predicts the breaking of the C–O bond. In addition, at 1200-1280cm^{-1}, we deconvoluted the generated absorption signal, and the signal corresponding to -O(CO)O- changed more drastically than that of Ar-OH. This implies the preferential degradation of -O(CO)O-.

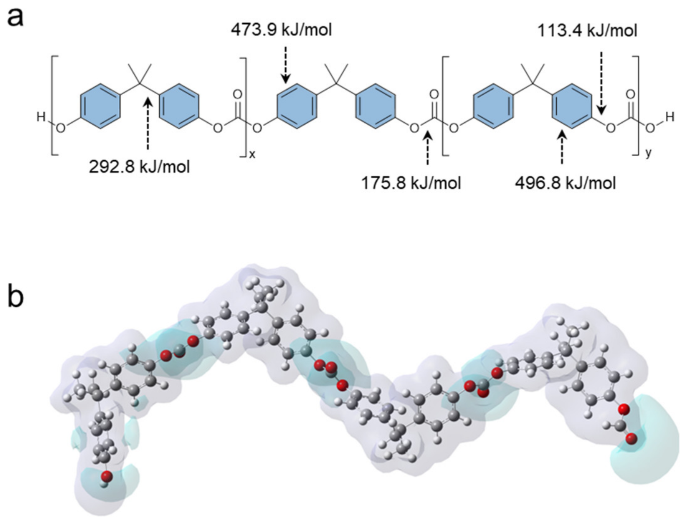

Supplementary Figure 31. The analysis of PC polymer. (a) Calculated bond energy for PC molecules (x=1, y=2 for approximation). The bond energies are extrapolated using the Laplace bond order scale after calculating the bond energies of the C-O bonds in the model at the m06x/6-311+G* level[7]. (b) Computing the electrostatic potential for a polycarbonate molecule with a polymerization degree of 4, where green denotes negatively charged areas and gray indicates positively charged zones.

The bond energy for PC molecule is calculated: C_{arom} -C_{aliph} bond energy is 292.8 kJ mol^{-1}, while aromatic ring bond energies reach 473.9 kJ mol^{-1} and 496.8 kJ mol^{-1}. The relatively low bond energies of C–O bonds (175.8 kJ mol^{-1} and 113.4 kJ mol^{-1} for C_{ester} -O and C_{arom} -O, respectively) in the whole system make it easy to form soluble BPA monomers. Electrostatic potential calculations reveal the carbonyl group as a negatively charged region, suggesting its potential reactivity within the PC structure. This finding implies that the carbonyl group may serves as a highly reactive site in the PC polymer.

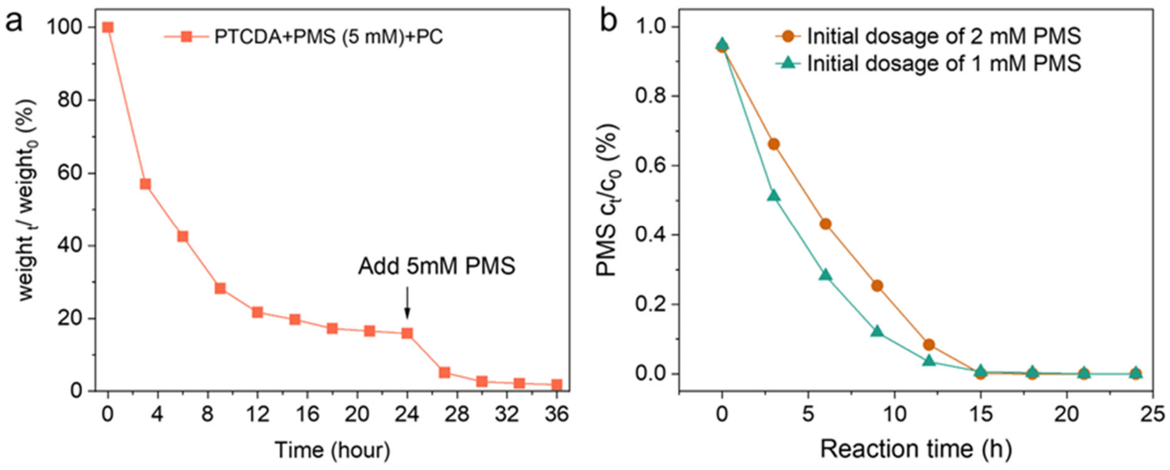

Supplementary Figure 32. The change of (a)PC microplastics mass and (b) PMS concentration during photocatalytic degradation of microplastics by PMS/PTCDA.

Upon the addition of 5 mM PMS, PC microplastic weight remained stable at 24 h with a loss of 84.1%. Subsequent introduction of 5 mM PMS led to a continuous decline in weight, culminating in complete decomposition at 36 hours. This outcome also highlights the stability of the PTCDA photocatalyst.

The residual PMS concentration was ascertained via colorimetric analysis. A color-developing solution was prepared, consisting of 0.5 g $NaHCO_3$, 10 g KI, and 100 mL deionized water. In standard tests, 1 mL of diluted filtrate was combined with 1 mL of the color-developing solution at specified intervals. After 15 minutes, PMS concentration was determined using a UV-Vis spectrophotometer at 400 nm.

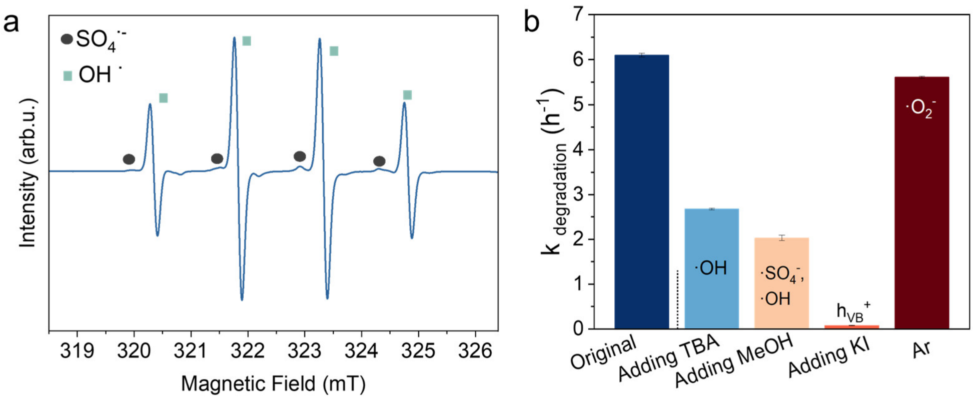

Supplementary Figure 33. Identification of active species during degradation. (a) Electron paramagnetic resonance (EPR) signal of PTCDA aqueous dispersion with or without PMS under in-situ irradiation (λ≥420 nm). (b) Effect of active species scavengers on the degradation rate of BPA. c Concentration of BPA and TOC curves in PTCDA photocatalytic oxidation BPA reaction. The error bars represent the standard deviation after three individual experiments.

PMS captures electrons resulting in the formation of free radicals, which can be demonstrated by in situ electron paramagnetic resonance (EPR) experiments with 5, 5-dimethyl-1-pyrroline N-oxide (DMPO). Only the DMPO-·OH adduct 1:2:2:1 ($A_{H\beta}=A_N=14.86G$) appeared under for PTCDA. The addition of PMS significantly enhanced the DMPO-·OH signal and an obvious DMPO-$SO_4^{\cdot-}$ adduct ($A_N=14.86G$, $A_{H\alpha}=12.90G$, $A_{H\beta}=2.35G$, $A_{H\gamma}=0.98G$) is discovered at the same time[8, 9]. This suggests that the enhanced electron density of PTCDA resulted in increased generation of the three active species.

The types of reactive species in the PMS/PTCDA system were determined by quenching experiments. Tert-butyl alcohol (TBA) was adopted as ·OH scavenger. MeOH as a scavenger for ·OH and ${}^{\cdot}SO_4^{-}$ [10, 11], and h^+ was quenched by KI [12]. Reaction in Argon atmosphere to identify the role of $\cdot O_2^{-}$. A significant decrease in photocatalytic degradation efficacy occurred after quenching of holes, ·OH and ${}^{\cdot}SO_4^{-}$, indicating that all three active substances contributed to the

photocatalytic degradation of organic pollutants.

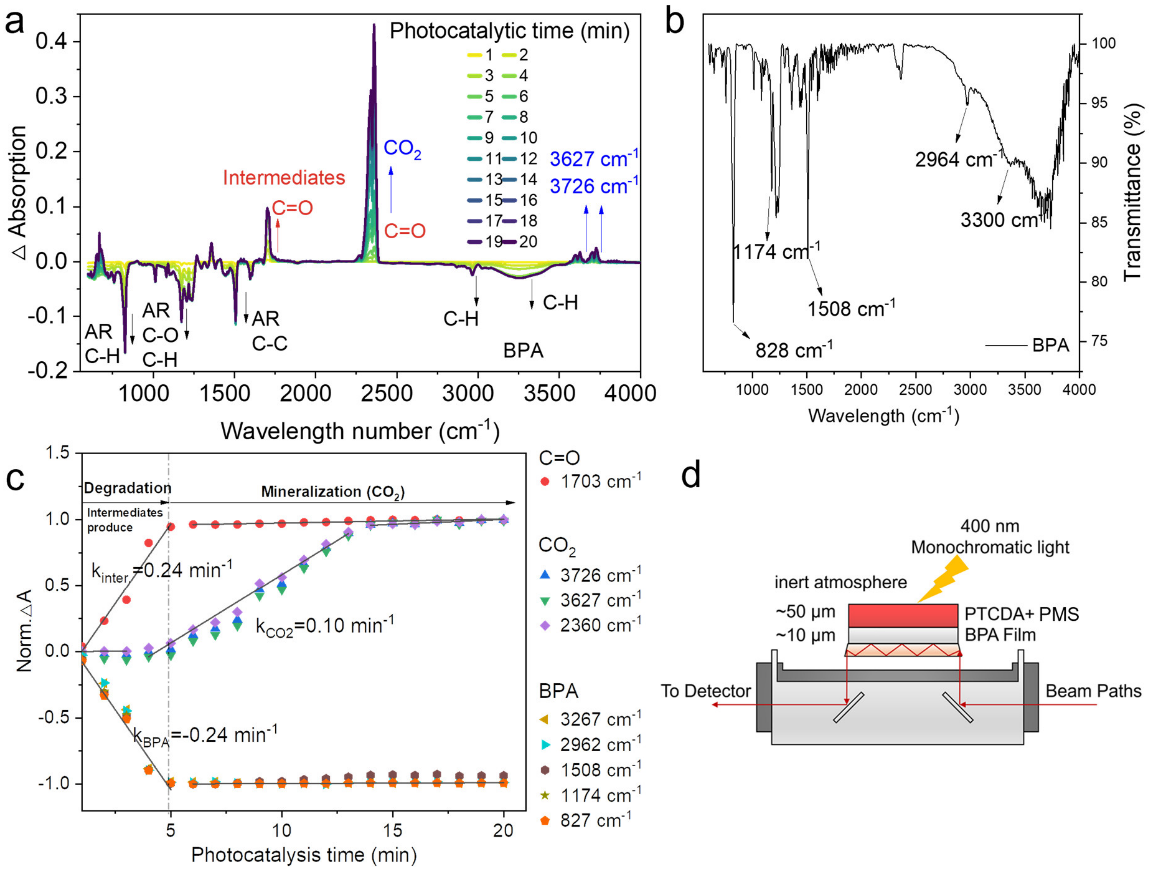

Supplementary Figure 34. Attenuated total reflection Fourier transform infrared spectroscopy (ATR-FTIR) analysis of the BPA mineralization. (a) FTIR of BPA powder; (b) ATR-FTIR in situ monitoring of the absorption spectrum of PMS/PTCDA photocatalytic degradation of BPA; (c)Representation of the absorption of CO_2, BPA and intermediates over time. (d) Schematic diagram of the ATR-FTIR in situ testing process.

The photocatalytic degradation of BPA by PMS/PTCDA was observed by ATR-FTIR differential absorption spectroscopy. 2360 cm^{-1} is the asymmetric expansion of CO_2. 3726, 3627 cm^{-1}, represents the vibration of gaseous CO_2. These characteristic peaks of CO_2 significantly increased. Based on the FTIR of the pure BPA tested, 3267, 2962, 1508, 1174, 827 cm^{-1} are the characteristic peaks of BPA. Where 3267 and 2962 cm^{-1} are attributed to the C-H vibration of methyl. 1508 is

attributed to the C-C vibration of aromatic rings; 1174 cm^{-1} is attributed to the O-H deformation and C-O stretching vibration interaction in aromatic rings; and 827 cm^{-1} is the C-H vibration of aromatic rings. At the same time, changes were also observed in the 1703 cm^{-1} absorption peaks related C=O stretching vibrations. The changes in the absorption peaks of these three substances over time in Fig.S24c showed discernible degradation and mineralization processes. And with 2360, 827, 1703 cm^{-1} for CO_2, BPA and intermediates respectively, the reaction rate constants were further fitted by linear equations, yielding a rate constant of -0.24 min^{-1} for the BPA reaction and 0.24 min^{-1} for the intermediate product production. Fig.S24d exhibits the test principles of in-situ ATR-FTIR.

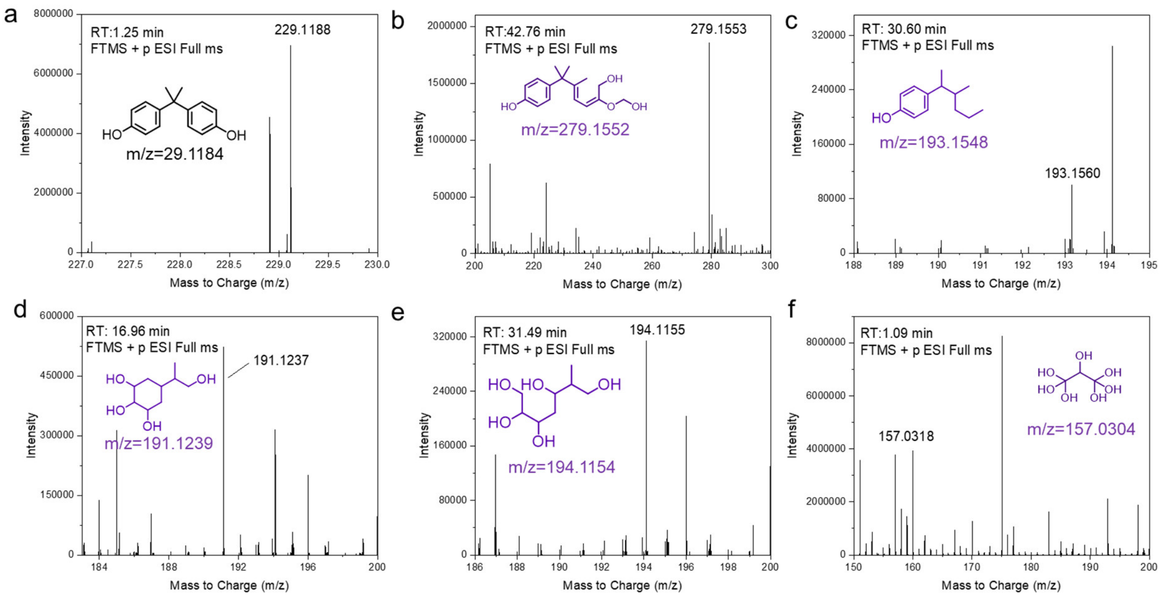

Supplementary Figure 35. Mass spectra of supernatants from photocatalytic decomposition of PC microplastics by PTCDA.

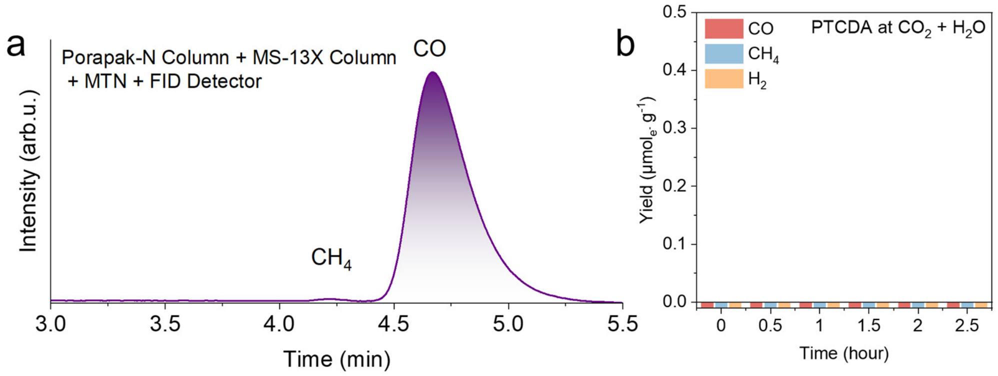

Supplementary Figure 36. Gaseous products of PTCDA (a) photocatalytic degradation of PC microplastics and (b) photocatalytic reduction of CO_2 tested by Shimadzu gas chromatography (GC-2014)

PTCDA is almost inert in CO_2 reduction under humid CO_2 atmosphere. (Test conditions: 10 mg PTCDA, 99.9999% CO_2, humidity 100%. Irradiation intensity: ~ 1Sun.) Taking into account the enhanced stability of PTCDA, the tested CO and CH_4 shows that the gas phase products generated originate from plastics rather than CO_2 reduction.

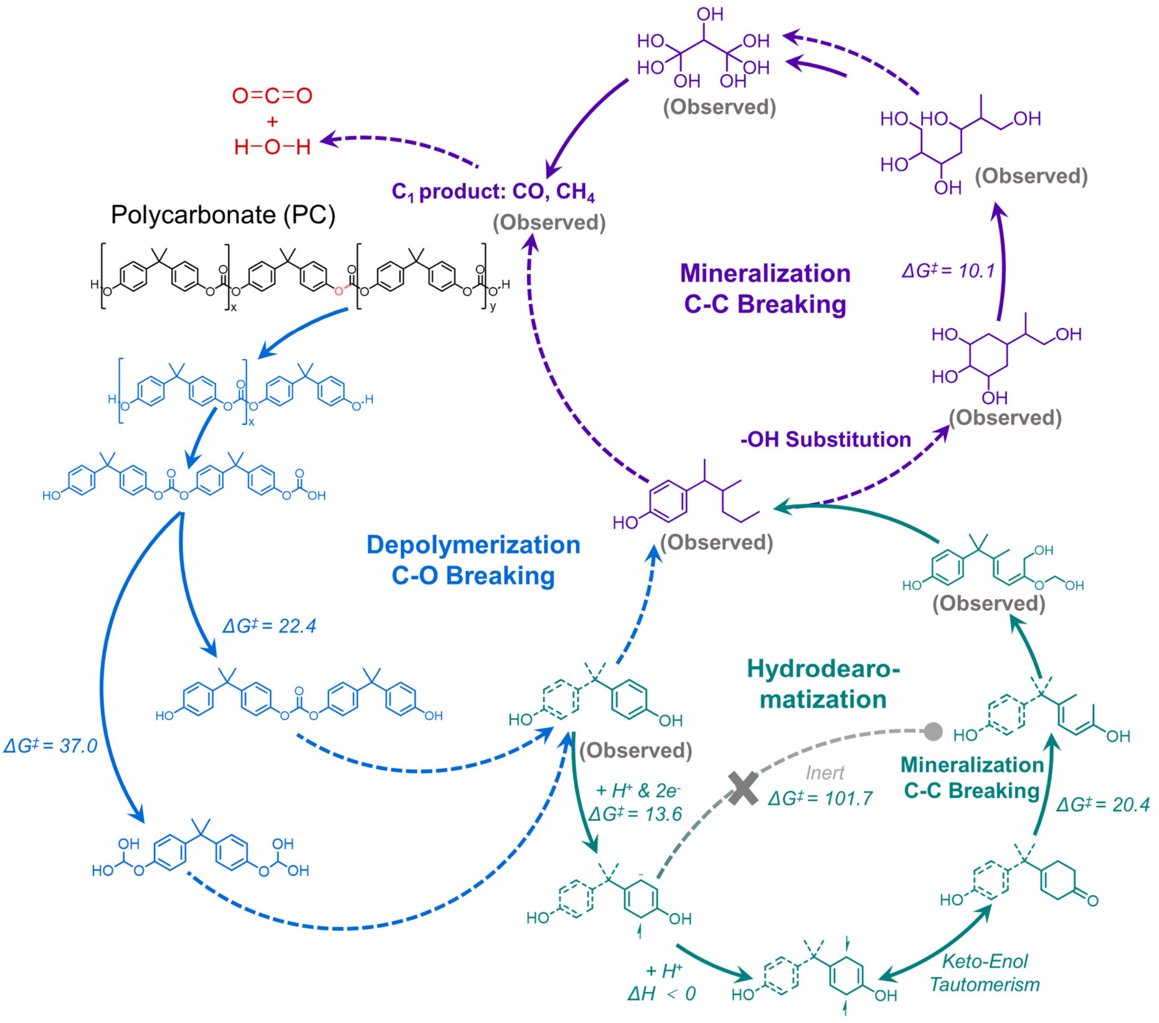

Supplementary Figure 37. Schematic diagram of decomposition and oxidation pathway analysis of PC microplastics according to mass spectrometry and DFT calculations. In typical calculations, for PC polymers, both x and y are taken as 2 for a reasonable simplification of the model. The unit of $\Delta G^{\ddagger}$ is kcal mol^{-1}.

To elucidate the PC microplastic decomposition and oxidation pathway, mass spectrometry and gas chromatography analysis were conducted on the supernatant and gas product, respectively. The energy rationalization of the transition state was also performed using DFT for the pathway presumed. A schematic diagram presents the inferred oxidation pathways deduced from the observed products. The pentameric molecule forms a dimer molecule via two C-O bond breaking steps, which then generates a monomeric BPA derivative ($\Delta G^{\ddagger}$=37.0 kcal mol^{-1}) and/or undergoes decarboxylation ($\Delta G^{\ddagger}$=22.4 kcal mol^{-1}). The ground state aromatic structure of BPA and its

derivatives remains stable due to a substantial energy barrier ($\Delta G^{‡}$ = 101.7 kcal mol^{-1}) for direct destruction, causing an inert step. We propose a proton-coupled electron transfer process in which BPA first receives a proton and two electrons to produce a carbon-negative center ($\Delta G^{‡}$=13.6 kcal mol^{-1}), followed by the spontaneous reception of a proton for conversion to the p-diene ring (ΔH ＜0). This ring subsequently undergoes Keto-Enol interchange, ring-opening ($\Delta G^{‡}$=20.4 kcal mol^{-1}), and multistep hydrodearomatization until full saturation achieved. Then the BPA derivative forms a dearomatized structure and undergoes a C-C cleavage with activation energies $\Delta G^{‡}$ of 10.1 kcal mol^{-1}, yielding the ring opening product. Subsequent multistep C-C breaks further generates small molecules with nearly saturated hydroxyl substituents. The final cleavage produces gaseous C_1 products which are then oxidized to form CO_2 and H_2O.

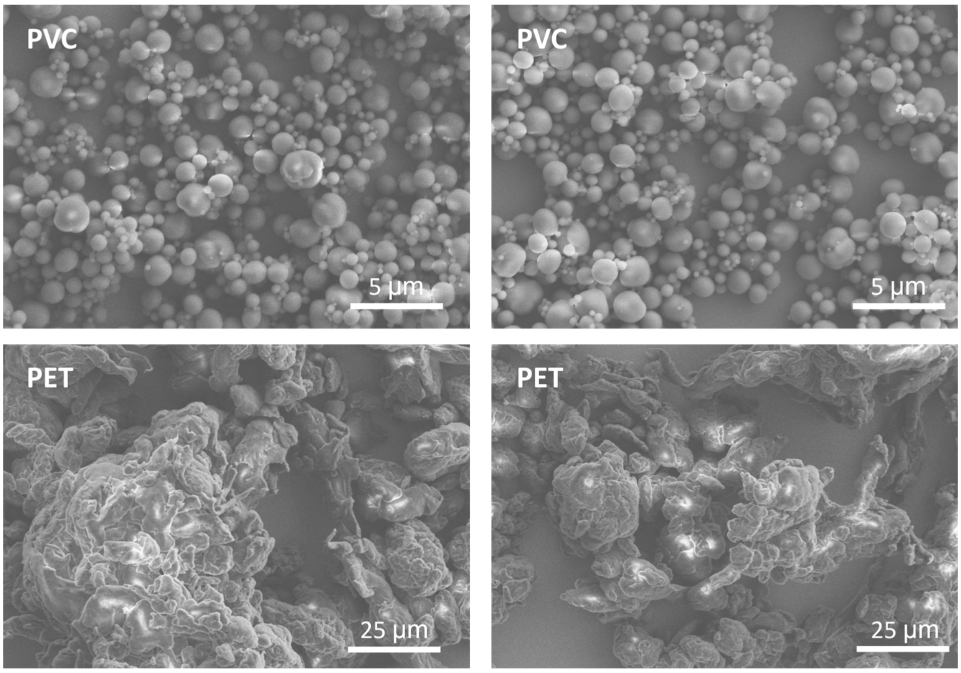

Supplementary Figure 38. SEM images PET and PC microplastics used in this work.

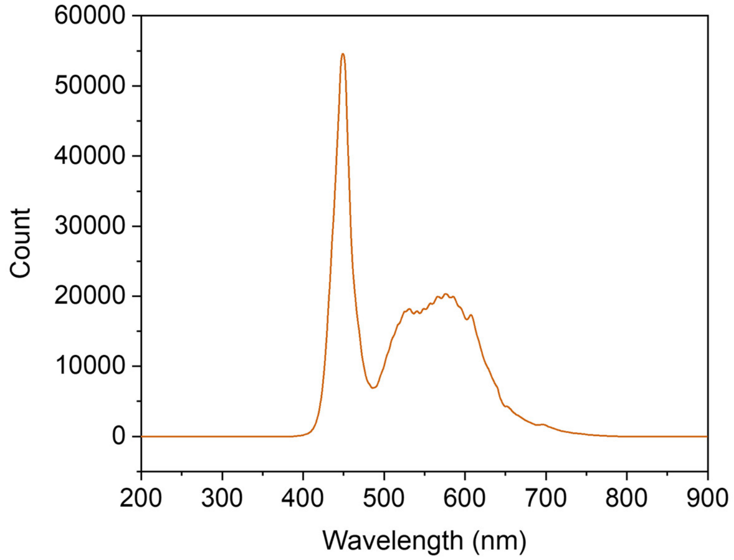

Supplementary Figure 39. Spectrogram used in this work

Supplementary Note 1: Interaction region indicator (IRI) functional analysis

The IRI functional analysis, a method predicated on electron density and its gradient, elucidates chemical bonds and weak interactions within molecules. Utilizing IRI facilitates an intuitive exploration of bonding characteristics and intermolecular forces, such as van der Waals and hydrogen bonds.

IRI is simply defined as follows [13]:

$$IRI(r) = \frac{|\nabla\rho(r)|}{[\rho(r)]^{a}} \quad (1)$$

An adjustable parameter 'a', is utilized, where a standard IRI definition requires a = 1.1. The IRI essentially represents a gradient norm of electron density, influenced by a scaled electron density function.

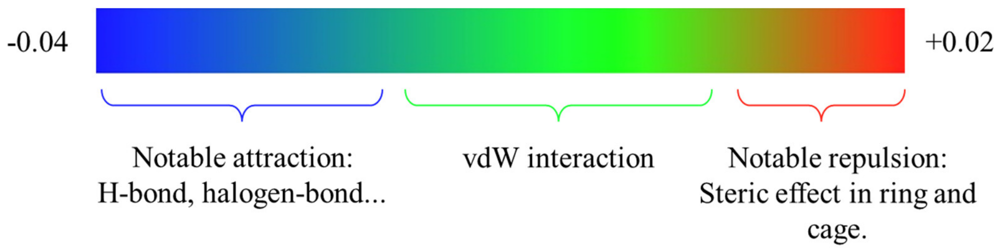

Supplementary Figure 40. Standard coloring method and chemical explanation of $sign(\lambda_2)\rho$ on IRI isosurfaces.

Supplementary Note 2: Principle of measuring surface potential with a kelvin probe force microscope (KPFM)

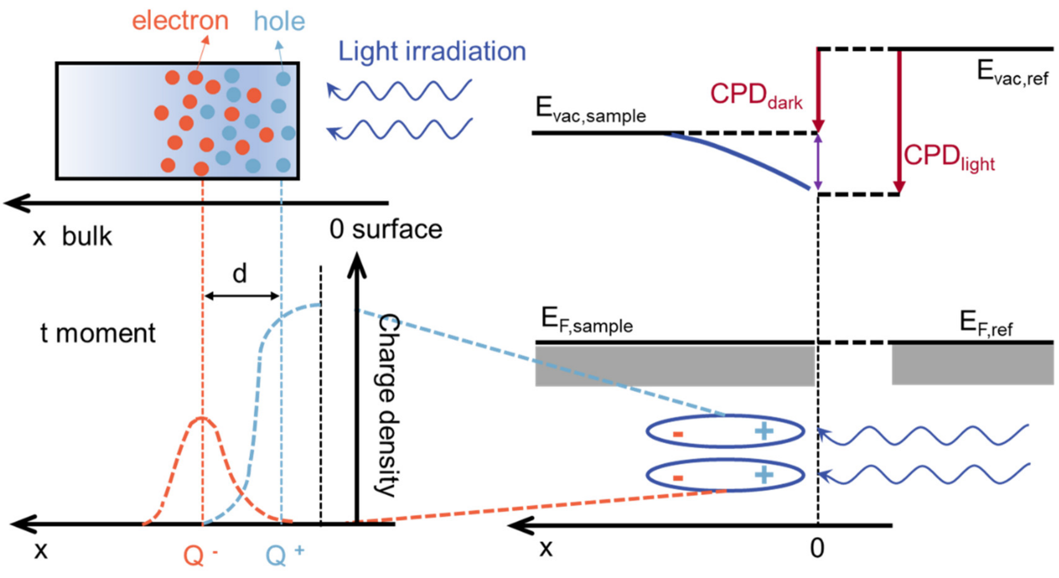

Supplementary Figure 41. Principle of KPFM for testing surface charge distribution of samples.

The space-time resolved surface potential originate from the contact potential difference (CPD) between the tip and the sample, further the photoinduced surface potential reflects the accumulation of photogenerated charges on the surface[14-16]. The surface photovoltage spectrum is quantified on the basis of Equation 2:

$$SPV = \frac{e}{\varepsilon\varepsilon_0} \times Q(t) \times d(t) = CPD_{light} - CPD_{dark} \quad (2)$$

In this work, highly oriented pyrolytic graphite (HOPG) is used as a substrate. Due to the negative electrical properties of the PTCDA surface, 1 mol L^{-1} of Zn^{2+} ($ZnCl_2$) was used to modify the HOPG in order to adsorb PTCDA. To test PTCDA adsorbed with PMS, PTCDA was dispersed in an aqueous solution containing PMS and dropped on the HOPG substrate. After drying, the PMS adsorbed on the substrate was washed off with a micro-water stream to obtain PTCDA adsorbed PMS on the surface.

Supplementary Note 3: The TOC analysis

The TOC is analyzed by Total Organic Carbon analyzer (Total Organic Carbon, referred to as TOC). TOC analysis tests the mechanism of organic pollutants: the water sample to be analyzed through high temperature combustion tube (900°C) by high temperature catalytic oxidation, so that organic compounds and inorganic carbonate are converted into carbon dioxide. After the acidification of the water sample in the reaction tube, the non-polar carbonate is decomposed into carbon dioxide, and the carbon dioxide generated is introduced into the non-dispersive infrared detector in turn, so that the total carbon and inorganic carbon in the water can be measured respectively. The difference between the two is the total organic carbon (TOC). That is, TOC analysis tests the conversion of undegraded organic pollutants into CO_2.

Supplementary Note 4: Continuous flow reactors

The 0.5 m^2 continuous flow reactor consists of four parts: water balance tank, photocatalytic reaction chamber, liquid detection, and liquid collection system. The water balance tank consists of a mixing tank and a reservoir--the length × width × height = 610 × 110 × 55 mm^3. Nine galleries compose the photocatalytic reaction cell: width of 40, 50, 60, 60, 80, 80, 100, 100, and 100 mm, a length of 610 mm, and a height of 30 mm. High concentrations of contaminants and PMS and tap water are thoroughly mixed by being pumped into the mixing cell. A peristaltic pump regulates the influent flow and initial concentration. The effluent flows into the reservoir to stabilize the water column and then flows into the photocatalytic reaction chamber through an overflow weir with oxygen aeration. The bottom of each runner layer is covered with a non-woven substrate that firmly supports the PTCDA. The water flow is zigzagged along the flow channel at an angle of 180° to obtain a long residence time while improving the mass transfer efficiency between the catalyst and the liquid. The concentration of the purified wastewater is detected in real-time by an absorption spectrum detector. Finally, all the wastewater enters the collection system to obtain purified water.

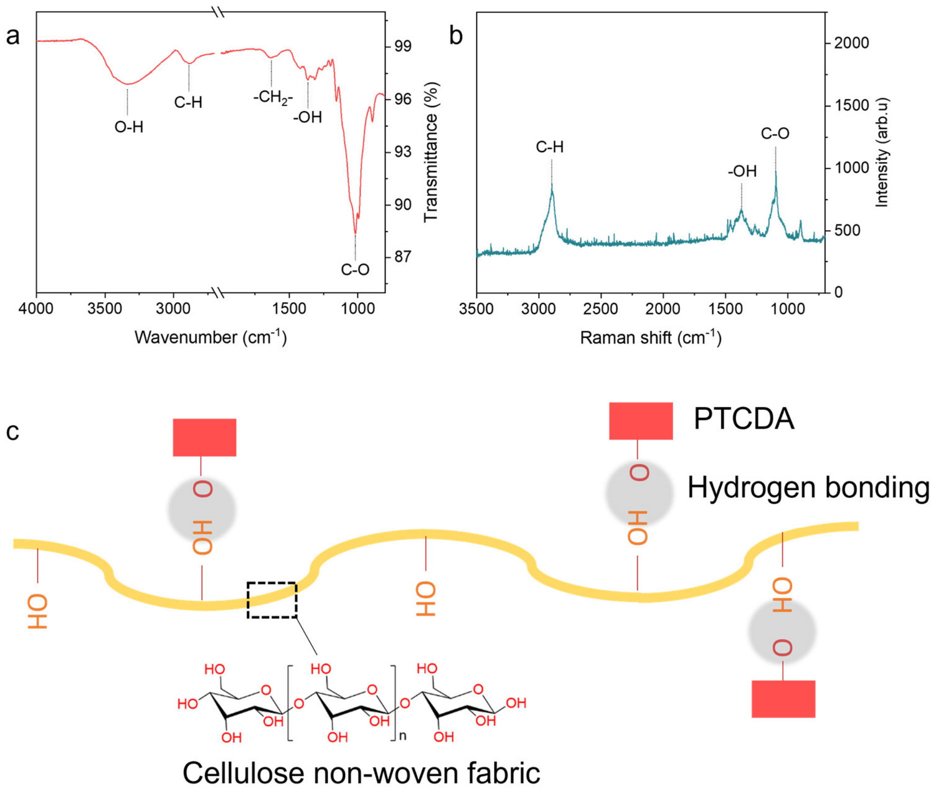

Supplementary Figure 42. Chemical structure of cellulose-based nonwovens. (a) FTIR spectrum, (b) Raman spectrum of cellulose nonwoven fibers. (c) Potential hydrogen bonding mode between cellulose nonwoven fibers and PTCDA.

Cellulose nonwovens were used as a substrate to support the PTCDA powder. The chemical structure of the cellulose was characterized by FTIR and Raman. FTIR spectra observed C-O (1017 cm^{-1}), -OH (1367 cm^{-1}), $-CH_2-$ (1634 cm^{-1}), C-H (2886 cm^{-1}), O-H (3339 cm^{-1}) peaks. OH (1370 cm^{-1}), C-H (2898 cm^{-1}) peaks.

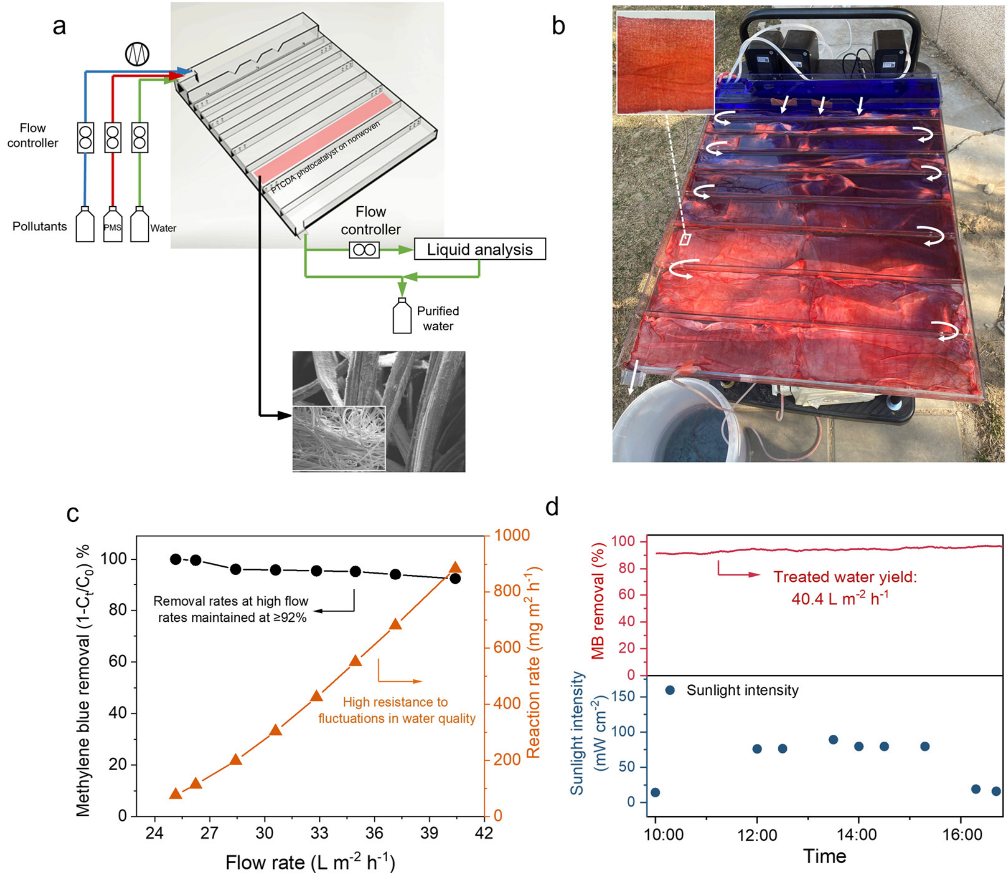

Supplementary Figure 43. (a) Schematic diagram of continuous-flow reactor for photocatalytic oxidation reaction. Bottom： SEM image of non-woven fibers loaded with PTCDA. (b) Photograph of photocatalytic degradation of methylene blue (MB) by PMS/PTCDA in a continuous flow reactor. The inset is an enlargement of the non-woven fabric loaded with PTCDA. Arrows indicate the direction of water flow. (c) MB removal rate and photocatalytic reaction rate in scalable continuous-flow reactor under simulated sunlight exposure indoors. c[cat.] =0.2 g m^{-2}. (d) Effect of MB removal under natural sunlight irradiation outdoors.

Supplementary Table 1. Simulated cell parameters of PTCDA molecular crystals

Lattice type	a (Å)	b (Å)	c (Å)	α (°)	β (°)	γ (°)
Monoclinic	4.500	14.700	12.000	90.000	91.480	90.000

Supplementary Table 2. Fitting parameters for the relationship between the electronic quantity and $\{S_{(0\ 1\ 1)}\}/\ \{S_{(1\ 1\ \bar{2})}\}$ in Figure 2a.

	Equation	k	b	R2
Electrons in PTCDA	y=kx+b	2.5×10^{10}	1.6×10^{11}	0.94
Holes in PMS/PTCDA		9.3×10^{10}	5.2×10^{11}	0.98

Supplementary Table 3. Parameters for fitting the decay of photogenerated electrons in transient absorption spectra

PTCDA		PMS/PTCDA	
A_1	A_2	A_1	A_2
0.030	0.510	2.007	0.510
τ_1 (ps)	τ_2 (ps)	τ_1 (ps)	τ_2 (ps)
5.69	284.87	2.69	96.90

* Exponential decay is fitted according to the following Equation 3:

$$y(x)=\sum_{i=1} A_i e^{\frac{x-x_0}{\tau_i}} \quad (3)$$

Supplementary Table 4. Parameters for fitting the decay of photogenerated holes in transient absorption spectra

PTCDA		PMS/PTCDA	
A_1	A_2	A_1	A_2
0.196	0.422	0.196	0.422
τ_1 (ps)	τ_2 (ps)	τ_1 (ps)	τ_2 (ps)
1.26	14.41	9.08	74.63

* Exponential decay is fitted according to the Equation 3.

Supplementary Table 5. Fitting parameter of absorption peak intensity in ATR-FTIR representing the variations of BPA and intermediates concentration

Time (min)	BPA(827 cm^{-1})			Intermediates(1703 cm^{-1})		
	k	b	R2	k	b	R2
1-5	-0.24	0.16	0.97	0.24	-0.23	0.96
5-20	6.112E-4	-1.002	0.71	0.003	0.940	0.85

* Fitting through linear Equation 4:

$$y(x)=kx+b \quad (4)$$

Supplementary Table 6. Fitting parameter of absorption peak intensity in ATR-FTIR representing the change of CO_2 concentration

Time (min)	CO_2 (2360 cm^{-1})		
	k	b	R2
1-4	9.734E-4	-6.180E-5	0.87
4-14	0.102	-0.440	0.98
14-20	0.007	0.854	0.87

* Fitting through linear Equation 4.

Supplementary Table 7. Qinghe River parameters

TOC[a] (mg L^{-1})	TN[b] (mg L^{-1})	AN[c] (mg L^{-1})	SO_4^{2-} (mg L^{-1})	Cl^- (mg L^{-1})	NO_3^- (mg L^{-1})
15.4	1.53	0.40	14.25	9.07	4.18

[a] TOC: total organic carbon

[b] TN: total nitrogen

[c] AN: ammonia nitrogen

[d] Water samples were filtered by the 0.45 μm membrane filtration to remove the particulate matters.

Supplementary References

1. Foley, D. J.; Coleman, S. P.; Tschopp, M. A.; Tucker, G. J., Correlating deformation mechanisms with X-ray diffraction phenomena in nanocrystalline metals using atomistic simulations. *Computational Materials Science* **2018,** *154*, 178-186.
2. Shi, Y.; Li, J.; Mao, C.; Liu, S.; Wang, X.; Liu, X.; Zhao, S.; Liu, X.; Huang, Y.; Zhang, L., Van Der Waals gap-rich BiOCl atomic layers realizing efficient, pure-water CO2-to-CO photocatalysis. *Nat Commun* **2021,** *12* (1), 5923.
3. Li, C.; Liu, J.; Li, H.; Wu, K.; Wang, J.; Yang, Q., Covalent organic frameworks with high quantum efficiency in sacrificial photocatalytic hydrogen evolution. *Nat Commun* **2022,** *13* (1), 2357.
4. Vdović, S.; Wang, Y.; Li, B.; Qiu, M.; Wang, X.; Guo, Q.; Xia, A., Excited state dynamics of β-carotene studied by means of transient absorption spectroscopy and multivariate curve resolution alternating least-squares analysis. *Phys. Chem. Chem. Phys.* **2013,** *15* (46), 20026-20036.
5. Chen, X. J.; Wang, J.; Chai, Y. Q.; Zhang, Z. J.; Zhu, Y. F., Efficient Photocatalytic Overall Water Splitting Induced by the Giant Internal Electric Field of a g-C3N4/rGO/PDIP Z-Scheme Heterojunction. *Adv. Mater.* **2021,** *33* (7), 7.
6. He, X.; Zhu, G.; Yang, J.; Chang, H.; Meng, Q.; Zhao, H.; Zhou, X.; Yue, S.; Wang, Z.; Shi, J.; Gu, L.; Yan, D.; Weng, Y., Photogenerated Intrinsic Free Carriers in Small-molecule Organic Semiconductors Visualized by Ultrafast Spectroscopy. *Sci. Rep.* **2015,** *5* (1), 17076.
7. Lu, T.; Chen, F., Bond Order Analysis Based on the Laplacian of Electron Density in Fuzzy Overlap Space. *The Journal of Physical Chemistry A* **2013,** *117* (14), 3100-3108.
8. Liu, G.; You, S.; Tan, Y.; Ren, N., In Situ Photochemical Activation of Sulfate for Enhanced Degradation of Organic Pollutants in Water. *Environ. Sci. Technol.* **2017,** *51* (4), 2339-2346.
9. Duan, X.; Sun, H.; Wang, Y.; Kang, J.; Wang, S., N-Doping-Induced Nonradical Reaction on Single-Walled Carbon Nanotubes for Catalytic Phenol Oxidation. *ACS Catal.* **2015,** *5* (2), 553-559.
10. Buxton, G. V.; Greenstock, C. L.; Helman, W. P.; Ross, A. B., Critical Review of Rate Constants for Reactions of Hydrated Electrons, Hydrogen Atoms and Hydroxyl Radicals (OH/O) in Aqueous Solution. *J. Phys. Chem. Ref. Data* **1988,** *17* (2), 513-886.
11. Neta, P.; Huie, R. E.; Ross, A. B., Rate Constants for Reactions of Inorganic Radicals in Aqueous Solution. *Journal of Physical and Chemical Reference Data* **1988,** *17* (3), 1027-1284.
12. Guo, Y.; Nan, J.; Xu, Y.; Cui, F.; Shi, W.; Zhu, Y., Thermodynamic and dynamic dual regulation $Bi_2O_2CO_3/Bi_5O_7I$ enabling high-flux photogenerated charge migration for enhanced visible-light-driven photocatalysis. *J. Mater. Chem. A* **2020,** *8* (20), 8.
13. Lu, T.; Chen, Q., Interaction Region Indicator: A Simple Real Space Function Clearly Revealing Both Chemical Bonds and Weak Interactions**. *Chemistry–Methods* **2021,** *1* (5), 231-239.
14. Nonnenmacher, M.; Oboyle, M. P.; Wickramasinghe, H. K., KELVIN PROBE FORCE MICROSCOPY. *Applied Physics Letters* **1991,** *58* (25), 2921-2923.
15. Melitz, W.; Shen, J.; Kummel, A. C.; Lee, S., Kelvin probe force microscopy and its application. *Surface Science Reports* **2011,** *66* (1), 1-27.

16. Cui, P.; Wei, D.; Ji, J.; Huang, H.; Jia, E.; Dou, S.; Wang, T.; Wang, W.; Li, M., Planar p–n homojunction perovskite solar cells with efficiency exceeding 21.3%. *Nature Energy* **2019,** *4* (2), 150-159.
